# The Use of High-Throughput Phenotyping for Assessment of Heat Stress-Induced Changes in Arabidopsis

**DOI:** 10.34133/2020/3723916

**Published:** 2020-07-17

**Authors:** Ge Gao, Mark A. Tester, Magdalena M. Julkowska

**Affiliations:** Division of Biological and Environmental Sciences and Engineering (BESE), King Abdullah University of Science and Technology (KAUST), Thuwal 23955-6900, Saudi Arabia

## Abstract

The worldwide rise in heatwave frequency poses a threat to plant survival and productivity. Determining the new marker phenotypes that show reproducible response to heat stress and contribute to heat stress tolerance is becoming a priority. In this study, we describe a protocol focusing on the daily changes in plant morphology and photosynthetic performance after exposure to heat stress using an automated noninvasive phenotyping system. Heat stress exposure resulted in an acute reduction of the quantum yield of photosystem II and increased leaf angle. In longer term, the exposure to heat also affected plant growth and morphology. By tracking the recovery period of the WT and mutants impaired in thermotolerance (*hsp101*), we observed that the difference in maximum quantum yield, quenching, rosette size, and morphology. By examining the correlation across the traits throughout time, we observed that early changes in photochemical quenching corresponded with the rosette size at later stages, which suggests the contribution of quenching to overall heat tolerance. We also determined that 6 h of heat stress provides the most informative insight in plant's responses to heat, as it shows a clear separation between treated and nontreated plants as well as the WT and *hsp101*. Our work streamlines future discoveries by providing an experimental protocol, data analysis pipeline, and new phenotypes that could be used as targets in thermotolerance screenings.

## 1. Introduction

Globally, the last decade was the warmest since 19th century and resulted in record-breaking heatwaves in many parts of the world [1, 2]. Heat stress leads to a reduction of plant performance and productivity at all developmental stages, making the heatwaves a serious threat to agriculture. However, the majority of the efforts in heat stress research focus on early seedling development, scoring survival, or hypocotyl elongation [3–5] or reproductive stages [6, 7], where the pollen viability is reduced by high temperatures. The handful of studies focusing on the heat stress responses at the vegetative development stage [8–10] show that heat tolerance at the vegetative stage contributes to resilience at the reproductive stage. Therefore, understanding the changes caused by heat stress and breeding for heat tolerance at all developmental stages is essential to ensure future sustainable food supply.

*Arabidopsis thaliana* has been widely used in screenings for thermotolerance, predominately focusing on seedling viability [11–13], hypocotyl elongation [14, 15], or seed germination [5] on agar plates. As heat tolerance relies on multiple processes, quantification of simple traits, determined by the ease of phenotyping rather than physiological importance, does not provide the best tools capturing the complexity of the responses, e.g., plant cooling capacity, growth recovery, and maintenance of photosynthesis, which all contribute to the diversity of thermotolerance mechanisms. Continuous monitoring of plant growth after heat exposure via nondestructive methods, such as RGB, thermal imaging, and chlorophyll fluorescence, provides insight into physiological responses corresponding to photosynthetic efficiency and plant cooling abilities which cannot be scored by the eye. The automated and environmentally controlled system enables time-efficient screening of large populations in a single experiment. Phenotypic traits, such as plant size, temperature, and photosynthetic efficiency have been successfully applied to evaluate plant performance under drought [16, 17], salinity [18], and chilling [17] stress. A recent study by (Chen et al., 2019) used high-throughput phenotyping to describe the physiological consequences of heat and drought stress at the early flowering stage of *Brassica rapa*. The maximum carboxylation rate allowed by Rubisco, rate of triose phosphate use, and flower volume were identified as traits associated with high stress tolerance. As measuring these traits requires specialized equipment (LiCOR) and is restricted to the flowering stage, there is still a need to examine heat stress responses at the vegetative stage, providing clues to germplasm selection as much shorter developmental timescale, using a high-throughput setup.

Heat stress disturbs cellular homeostasis, reducing plant growth and development, and in extreme cases can result in plant death. Heat exposure activates heat stress transcription factors, which induce the transcription of heat shock proteins (hsp). hsp acts as molecular chaperones (hsp70, hsp90, hsp60, hsp100 and, small hsps, [19]), binding to partially denatured proteins and preventing their irreversible inactivation and aggregation ([20]); hsp 101 is a small hsp, which localizes to stress granules during hear stress [21], where it plays an important role in acquired thermotolerance. hsp101 was one of the earliest genes identified to have a crucial role in thermotolerance in Arabidopsis, with no detrimental effects on normal growth or development in the absence of stress [22, 23]. Homologues of hsp101 were identified and characterized for their role in heat stress response in maize, soybean, wheat, tobacco and pea, and kidney bean (Keeler et al., 2000; Katiyar-Agarwal et al., 2001). As such, the *hsp101* mutant showed severe reduction in heat tolerance compared to WT in terms of survival; however, the broader knowledge about the processes compromised in this mutant during the heat exposure is underexplored.

In this study, we investigated the feasibility of applying RGB, kinetic chlorophyll fluorescence, and infrared imaging for evaluating heat stress response in Arabidopsis. We developed a physiologically relevant heat-imposition protocol for the vegetative stage of Arabidopsis plants based on the significant changes observed for multiple traits. Additionally, by studying the heat-induced changes in the WT and *hsp101* mutant, we were able to identify additional traits which might indicate compromised heat stress tolerance. By applying machine learning, we identified that maintenance of photochemical quenching immediately after stress application could be potentially used as an indicator for heat stress tolerance, as it corresponded with the increase in plant size at the later time points. This work provides a primer for future studies using high-throughput phenotyping platforms, uncovering novel components of heat stress tolerance.

## 2. Materials and Methods

### 2.1. Plant Materials and Growth Conditions

Seeds of Arabidopsis wild-type Col-0 (CS60000) and *hsp101* (AT1G74310; *hot 1-3*, NASC ID: N16284) were stratified for three days at 4°C in the dark and germinated in a controlled environment in the PSI growth room (Photon Systems Instruments, Czech Republic). The environmental setting of the PSI growth room was set at 22°C (sensor sensitivity range: ±0.1°C), with a relative humidity of 60% (sensor sensitivity range: ±1%) and 400 ppm (sensor sensitivity range: ±100 ppm) of CO_2_. At the four-leaf stage (day 14 after sowing), healthy seedlings with similar size were transferred into PSI standard pots (6cm × 6cm × 9.5cm) filled with 100 g (±1.0 g) of the growing mix (SunGro Horticulture Metro-Mix 360, MA, USA), placed into PSI trays (5 × 4 pots per tray) and registered into the PlantScreen™ system. All pots were automatically weighed and watered every day to reach and maintain the weight of 130 g. Plants were grown under cool-white LED panel with a 16 h/8 h light/dark cycle, the light intensity received at plant rosette level is ~150 *μ*mol m^−2^ s^−1^. All plants were kept in the PSI growth room during the experiment except during the heat treatment.

### 2.2. Heat Stress Treatment and Phenotyping Experimental Design

**Figure 1:**
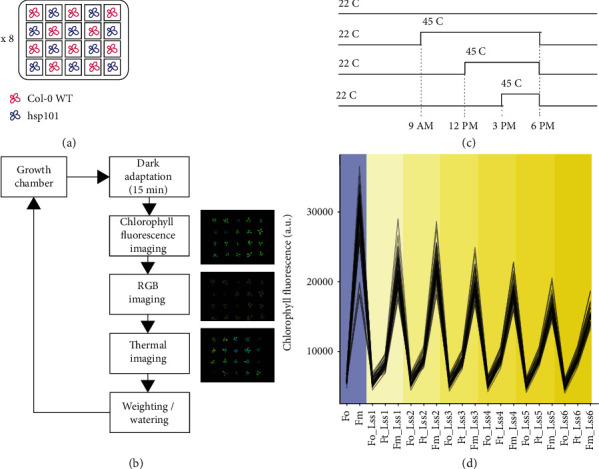
Schematic of the experimental setup. (a) WT and *hsp101* seedlings were grown in standardized pots supplied by the PlantScreen™ phenotyping system using a checkerboard design across 8 trays, with 10 WT and *hsp101* seedlings in each tray. The environment in the growth room was set to a 16/8 h day/night cycle, with 22°C and 60% relative humidity. (b) The phenotyping protocol. Each tray underwent an initial 15 min dark-adaptation period inside the adaptation chamber, followed by chlorophyll fluorescence, red green blue (RGB), and thermal imaging, with automatic weighing and watering before returning to the growth chamber. (c) The heat stress imposition protocol. 22 days after sowing, two trays of plants were kept in the growth chamber as control and other six trays were moved into the preheated 45°C Percival chamber at 9 am, 12 pm, and 3 pm for the 9 h, 6 h, and 3 h heat treatments, respectively. All treated six trays were returned to the growth chamber at 6 pm and imaged daily at 7 pm starting from the day before the heat stress application (DAS -1) until one week after. (d) The overview of the chlorophyll fluorescence protocol executed using the dark-adapted plants. The minimal (*F*_0_) and maximal (*F*_m_) fluorescence are measured directly after dark adaptation, followed by gradual exposure to increasing light intensities of 95, 210, 320, 440, 555, and 670 *μ*mol m^−2^ s^−1^, corresponding to Lss1, Lss2, Lss3, Lss4, Lss5, and Lss6, respectively, where the minimal (*F*_0_′) and steady-state fluorescence are determined. At each light intensity, plants are exposed to a saturating light flash, which allows measuring the maximum fluorescence at the light-adapted state for a given intensity (*F*_m_′).

At day 22 after sowing, we subjected plants to 3 h, 6 h, or 9 h of heat stress and control treatments (Figure 1). For each treatment group (3 h, 6 h, 9 h, or control), there are two trays each containing 10 WT and 10 *hsp101* plants next to each other in an evenly distributed design. Heat stress was applied by placing the 9 h, 6 h, and 3 h treatment trays into a preheated Percival growth chamber (Model CU36-L5, Percival Scientific, IA, USA) with white lights on (45°C, ~120 *μ*mol m^−2^ s^−1^) from 9 am to 6 pm, 12 pm to 6 pm, and 3 pm to 6 pm, respectively. Two trays were used as the “control treatment” with the same composition of genotypes remaining at the PSI growth room. After heat stress application, six trays were transferred back to the PSI growth room. The transfer of the trays between the phenotyping facility and the heated growth chamber lasted between 2 and 3 minutes. Plants were imaged using chlorophyll fluorescence, RGB, and infrared cameras daily at 7 PM starting from the day before the heat stress application (DAS -1) until one week after (Figure 1). Using an image-based analysis, we acquired a variety of traits reflecting plant growth, photosynthetic efficiency, rosette morphology, and temperature in the WT and *hsp101* plants and explored these phenotypes for phenotypic plasticity in response to heat stress and their feasibility for large thermotolerance screening. In total, we screened 160 individual plants, with 20 biological replicates per genotype per treatment, to develop an understanding of changes induced by treatment, genotype, and interaction between them for various traits.

### 2.3. Imaging-Based Phenotypic Measurements

Plant imaging was initiated one day before the heat application (DAS -1) to provide a baseline for the analysis and was performed daily at 7 pm until 7 days after the heat application (DAS 7). Each imaging round consisted of an initial 15 min dark-adaptation period inside the acclimation channel, followed by chlorophyll fluorescence, red green blue (RGB) coloured, and infrared (IR) imaging. For each imaging round, the phenotyping time for all trays was 80 mins, the trays were measured in the order of replicate tray 1 control-3 h-6 h-9 h and replicate tray 2 control-3 h-6 h-9 h. Lighting conditions, plant positioning, and camera settings were fixed throughout the experiment.

Chlorophyll fluorescence imaging unit in PlantScreen™ Systems constructed by Photon System Instruments (PSI, Czech Republic) measures the reemitted light approximating the photosynthetic performance of plants' photosystem II. The light curve protocol from PSI was applied to provide a detailed information on fluorescence kinetics during heat stress recovery ([24, 25]); a detailed protocol can be found in Figure S1. After 15 minutes of dark adaptation, an initial flash of light was applied to measure the minimum fluorescence (*F*_0_), followed by a saturation pulse to determine the maximum fluorescence (*F*_m_) in the dark-adapted state. Next, six 60 s intervals of cool-white actinic light with increasing intensity of 95, 210, 320, 440, 555, and 670 *μ*mol m^−2^ s^−1^ were applied to record the chlorophyll fluorescence signal at the end of each actinic light period as the steady-state fluorescence in the light-adapted state (*F*_t_′), followed by the signal measured at the saturation pulse as the maximal fluorescence in the light-adapted state (*F*_m_′). Based on those basic chlorophyll fluorescence signals, variable fluorescence during the dark-adapted state (*F*_v_, calculated as *F*_m_ − *F*_0_), the maximum quantum yield of PSII photochemistry (QY max calculated as *F*_v_/*F*_m_), variable fluorescence in a steady state (*F*_v_′, calculated as *F*_m_′–*F*_0_′), steady-state PSII quantum yield in the light-adapted state (QY′, calculated as (*F*_m_′–*F*_t_)/*F*_m_′), steady-state nonphotochemical quenching (NPQ, calculated as (*F*_m_–*F*_m_′)/F_m_′)), coefficient of nonphotochemical quenching during light-adapted state (qN, calculated as (*F*_m_ − *F*_m_′)/(*F*_m_ − *F*_0_′)), coefficient of photochemical quenching during light-adapted state (qP, calculated as (*F*_m_′ − *F*_t_)/(*F*_m_′ − *F*_0_′)) (Table S1).

Plant growth and morphological traits were obtained by RGB imaging unit using a 5-megapixel RGB camera (SV-0814H, VS Technology) mounted above the passing trays, providing the top-view image. The colour images were processed in PlantScreen™ Analyser software to develop plant masks, used for chlorophyll fluorescence imaging and extraction of morphological parameters such as rosette compactness, eccentricity, and roundness, calculated by the PlantScreen™ Analyser software (methods and individual traits are described in more detail in [18]). Thermal camera (A615, FLIR) was used to detected infrared radiation, which is converted into an electronic signal and subsequently processed to provide thermal images. The RGB mask was used to extract pixels belonging to individual plants on the thermal image and output the average surface temperature of the rosette area.

### 2.4. Data Analysis

Raw data were retrieved from the PlantScreen™ Analyser software, where the plant size, morphology, and chlorophyll fluorescence traits were preprocessed and calculated. The plants that did not grow or died during the experiments were removed from the data analysis, resulting in the final set of 1332 measurements of 148 individual plants across the 9-day imaging period. Data analysis was performed in R (version 4.0.0, http://www.r-project.org/), where the temporal changes across individual traits were visualized using the *ggplot2* (version 3.3.0 [26]) package and statistical analysis using *ggpubr* (version 0.3.0 [27]). The linear models examining the interaction between treatment and genotype were set up in R using the lm() function, as lm(trait ~ genotype + treatment + genotype : treatment) for each control and heat stress treatment separately. The correlation heatmaps were produced using the Pearson correlation with the package corrplot (version 0.84, [28]). Machine learning classification was implemented using Scikit-learn in Python [29]. The script used for the data analysis in R is publicly available as an R-notebook (10.5281/zenodo.3534239), as well as the Jupyter notebook containing the command lines used for machine learning (10.5281/zenodo.3534148).

## 3. Results

### 3.1. Extended Exposure to Heat Stress Results in Proportional Decrease of Rosette Size and Photosynthetic Efficiency

**Figure 2:**
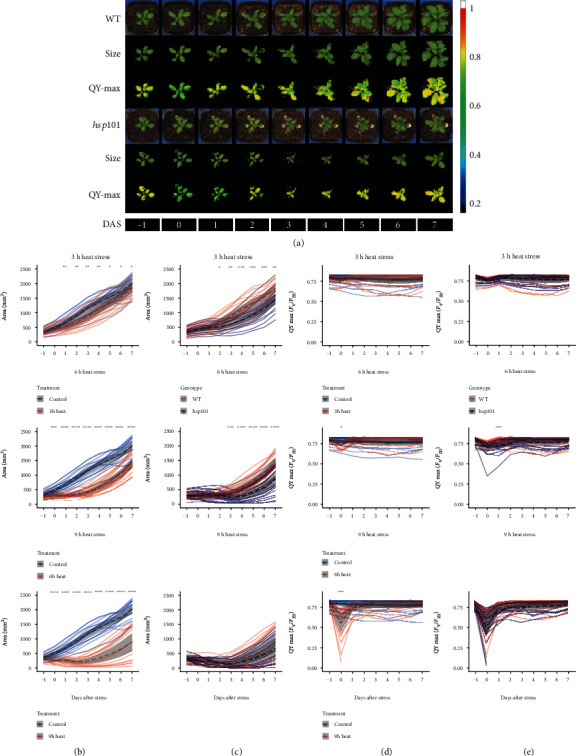
More pronounced reduction of the rosette size with increased length of heat treatment and mutation of hsp101. (a) Representative RGB, mask, and chlorophyll fluorescence images of WT and *hsp101* rosettes of plants underwent 6 h heat stress treatment. The colour code depicted at the right represents the maximal quantum yield (QY max) from blue (low *F*_v_/*F*_m_) to red (high *F*_v_/*F*_m_). DAS: days after stress. (b) Comparisons of rosette size of nonstressed WT plants with 3 h, 6 h, and 9 h heat-stressed WT. (c) Comparisons of rosette size of 3 h, 6 h, and 9 h heat-stressed WT and *hsp101*. (d) Comparisons of QY max of nonstressed WT plants with 3 h, 6 h, and 9 h heat-stressed WT. (e) Comparisons of QY max of 3 h, 6 h, and 9 h heat-stressed WT and *hsp101*. Dashed lines and grey ribbons represent the meanvalue ± 95%confidenceintervals of different plant groups (*n* varies between 17 and 20 for individual groups). The significance of the difference in the size of treated and nontreated WT (b, d) and *hsp101* (c, e) plants each day during the imaging period was determined by Student's *t*-test, with a *p* value below 0.05, 0.01, 0.001, and 0.0001 indicated with ^∗^, ^∗∗^, ^∗∗∗^, and ^∗∗∗∗^, respectively.

To assess whether high-throughput phenotyping can capture significant alterations in plant physiology caused by exposure to heat stress, we exposed three-week-old Arabidopsis plants to 3 h, 6 h, or 9 hours of acute heat stress (45°C) and evaluated the plant size, morphology, temperature, and chlorophyll fluorescence for subsequent 7 days after stress (DAS) imposition (Figure 1). All three heat stress treatments resulted in a significant reduction of the rosette size (Figure 2(b)), and these differences were substantial already 1 h after heat stress treatment. In general, the extended exposure to high temperature increased the effect observed on the rosette size (Figure 2(b)). The applied treatments were sublethal to the plants, as only four plants died after 9 h of exposure to heat stress, while other 36 plants that underwent this treatment were able to recover from the stress and produced new green leaves. These results suggest that soil-grown 3-week-old Arabidopsis plants are resilient to acute heat stress and that 6 h of heat stress treatment is best at differentiating between the WT and heat-sensitive mutant *hsp101*(Figure 2(c)).

To further evaluate the changes in the relationship to overall heat tolerance, we compared the WT and *hsp101* mutant (Figures 2(a) and 2(c)). After heat stress imposition, the *hsp101* plants developed smaller rosette sizes than WT (Figure 2(c)), while no significant difference in rosette size was observed between WT and hsp101 without heat stress imposition (Figure S1A). The significant differences in rosette size were observed between WT and *hsp101* two days after the 3 h heat treatment and three days after the 6 h treatment. No difference between WT and *hsp101* was observed after the 9 h treatment, where the rosette size was reduced in similar degree (Figure 2(c)). Significant interactions between the treatment and genotype were observed for the 3 h and 6 h treatments, while no interaction was observed for the 9 h treatment for rosette area (Table S2).

While plant size broadly reflects overall plant performance, high temperature can also influence plant morphology, such as the petiole elongation and increased leaf angle [30, 31]. We examined the effect of heat stress on rosette morphology and observed that heat stress treatment had a pronounced effect on rosette perimeter, compactness, and slenderness of leaves already one hour after heat stress application (Figure S2A). In addition, these three parameters showed significant differences between heat stress-treated WT and *hsp101* lines (Figure S2B). The significant interaction between the treatment and genotype was observed exclusively for the rosette perimeter at 6 h of heat stress treatment (Table S2). Another morphological change that we observed one day after the heat application was the increase in leaf angle, which occurred only in WT plants but not in *hsp101* (Figure S2D). The change in the leaf angle was reflected by the transient increase of rotational mass symmetry in WT at DAS 1, but returned back to levels observed in nonstressed plants 2 days after stress application (Figure S2B).

The decrease in plant photosynthetic efficiency is generally believed to precede developmental changes. Based on the chlorophyll fluorescence, we indeed observed a decline in maximum quantum yield (QY max), derived from the measurements of dark-adapted minimum (*F*_0_, Figure S3) and maximum fluorescence (*F*_m_, Figure S4) as (*F*_m_–*F*_0_)/*F*_m_. QY max indicates the efficiency of PSII photochemistry in the dark-adapted state and reveals the efficiency of electron transport inside PSII. An immediate decline in QY max occurred on the stress day (DAS 0) for both WT and *hsp101* (Figures 2(d) and 2(e)). The WT was able to recover the QY max 1 DAS to levels observed in nonstressed plants, whereas the QY max of *hsp101* plants recovered only at 2 DAS (Figure 2(e)). We also noted a severe reduction of the QY max in *hsp101* at the edges of the rosette (Figure 2(a)), which preceded the tissue senescence. The significant interaction between the treatment and genotype in QY max was observed for 6 h and 9 h of heat stress treatment (Table S2). The earlier reduction in QY max in these areas suggests that change in chlorophyll fluorescence can be used as early indicators of premature senescence.

**Figure 3:**
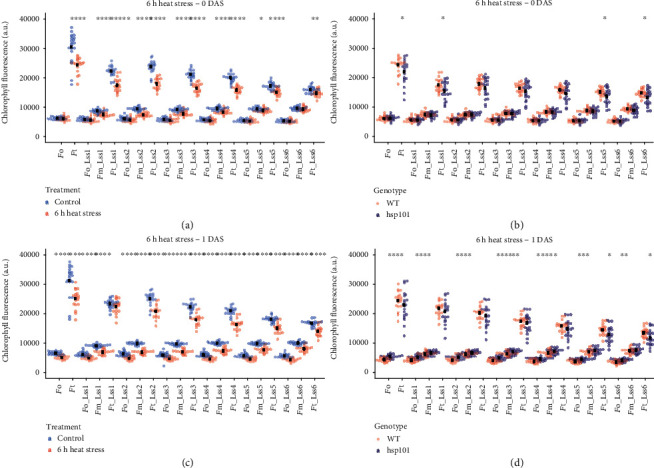
Heat stress-induced changes in chlorophyll fluorescence show a dynamic profile. The comparison of the directly measured chlorophyll fluorescence traits 1 h after 6 h of heat stress treatment (45°C) application in (a) WT compared to WT nonstressed plants and (b) WT compared to hsp101 plants exposed to heat stress. The directly measured chlorophyll fluorescence traits were also measured at 1 day after stress (DAS) in (c) WT compared to WT nonstressed plants and (d) WT compared to hsp101 plants exposed to heat stress. The average of individual groups is represented by the black dot, while the measurements derived from individual plants are represented using transparent points. The *F*_0_ and *F*_m_ indicate minimal and maximal chlorophyll fluorescence measured at dark-adapted state, respectively, while *F*_0_, *F*_t_, and *F*_m_ at Lss1 to Lss6 represent minimal, steady-state, and maximal chlorophyll fluorescence, respectively, in a light-adapted state at light intensities of 95, 210, 320, 440, and 670 *μ*mol m^−2^ s^−1^. The significance of the difference in chlorophyll fluorescence of treated and nontreated WT (a, c) and *hsp101* (b, d) plants for individual parameters were determined by Student's *t*-test, with a *p* value below 0.05, 0.01, 0.001, and 0.0001 indicated with ^∗^, ^∗∗^, ^∗∗∗^, and ^∗∗∗∗^, respectively.

Exposure to high temperature was earlier reported to result in an instant increase in minimal (*F*_0_) and decrease in maximum fluorescence (*F*_m_) in various plant species [32, 33]. The light-curve protocol used to examine the chlorophyll fluorescence, allowed us to study heat-induced changes in a light-adapted state at various light intensities (Figure 1(d)). Heat stress exposure resulted in lower *F*_m_ measured at 0 DAS (Figure S4), while the significant decrease for *F*_0_ was observed at 1 DAS in WT plants for all three heat stress regimes (Figure S3A). By examining all the directly measured traits at 0 DAS (Figure 3(a)), we noted a heat stress-induced decrease in light-adapted *F*_m_ at all studied light intensities. This reduction in maximum fluorescence was more pronounced in *hsp101* plants for the lowest and highest light intensities studied (Figure 3(b)). The heat stress also resulted in reduced minimal and steady-state fluorescence (*F*_t_) across studied light intensities at 1 DAS (Figure 3(c)). While 1 DAS, the *F*_m_ of heat-treated plants was still lower compared to nontreated plants, and the difference between WT and *hsp101* at 1 DAS was no longer significant (Figure 3(d)). These results exemplify the dynamics and rapid recovery of the chlorophyll fluorescence. The significant interaction between the genotype and treatment was observed for *F*_m_ measured at all light intensities for 6 h of heat stress treatment, while for 3 h of heat stress treatment, the significant interaction was observed for the light intensity of Lss3 and above (Table S2). These results indicate the usefulness of the light-curve protocol, which allows detecting stress-induced damage with varying sensitivity.

As plant's cooling capacity is expected to be affected by exposure to high temperature, we examined differences in leaf temperature recorded from infrared camera between heat-stressed and nonstressed plants (Figure S5). Heat exposure increased leaf temperature across three different heat stress treatments (Figure S5A), no significant differences in leaf temperature were observed between the WT and *hsp101* plants (Figure S5B), and no significant interaction between the genotype and treatment were detected at any stress level studied (Table S2).

### 3.2. Classification Model and Trait Selection to Differentiate Heat-Sensitive and Tolerant Lines

  As the high-throughput phenotyping dataset allows us to examine more than 80 phenotypes, it makes it difficult to rank individual phenotypes to select the best traits which allow clear differentiation of heat-sensitive genotypes. Therefore, we implemented machine learning to simultaneously explore all the quantitative phenotypes collected within our experiment to differentiate between the WT and *hsp101* mutant. We applied logistic regression with lasso regularization to select the most useful traits for classification. To identify the heat stress regime allowing us to differentiate between WT and *hsp101*, we compared the classification performance of phenotypic data from different treatment groups including rosette size, leaf temperature, and a subset of top morphological traits (perimeter, the slenderness of leaves, compactness, isotropy, and rotational mass symmetry) and chlorophyll fluorescence parameters measured in the dark-adapted state (*F*_0_, *F*_m_, and QY max). The accuracies of model prediction (Table 1) were modest, with the highest accuracy of 68.2% for the 3 h heat treatment. The predictions calculated for 9 h heat stress treatment were lower than for the nonstressed plants, implying that 9 h heat stress treatment was too severe and not suited to differentiate phenotypic differences between the genotypes. When we included all phenotypic traits in the model, including the chlorophyll fluorescence measured in the light-adapted state, we observed improved classification accuracies for all groups (Table 1). In line with our earlier analysis (Figure 2), the phenotypes recorded for plants treated with 6 h of heat stress provided the highest accuracy (81.5%) in differentiating between two genotypes. The top five contributing parameters to the classification model were related to temperature and morphology (compactness, isotropy, slenderness of leaves, and perimeter), suggesting that variability in morphological changes were most useful genotypic indicators during the overall imaging period. The chlorophyll fluorescence parameters determined to be the most indicative of the genotype are direct measurements of the chlorophyll fluorescence (*F*_t_′, *F*_0_, and *F*_0_′) and the photochemical quenching of the chlorophyll fluorescence at the light-adapted state (*F*_q_′). While some parameters were classified as good genotype indicators under all treatments, e.g., leaf temperature, we noticed that photochemical quenching at the second to highest light intensity (*F*_q_ Lss5) was unique to the 3 and 6 h of heat stress treatment. Although our logistic regression model performs well without accounting for temporal differences, we would like to recognize that increasing the number of replicates would allow us to set up models for individual time points, thereby fully integrating the temporal character of heat stress responses

### 3.3. Decline in Photochemical Quenching as an Early Indicator of Heat Stress Susceptibility

**Figure 4:**
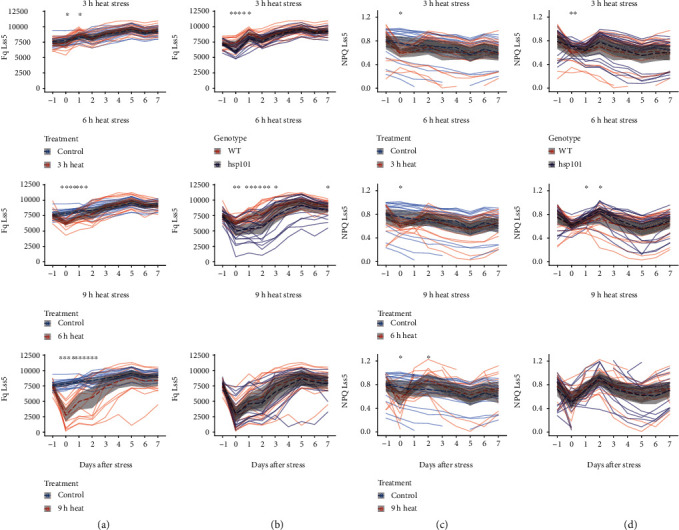
Heat stress transiently reduces photochemical and nonphotochemical quenching. (a) Comparison of photochemical quenching (*F*_q_) of WT plants grown under control conditions and 3 h, 6 h, and 9 h of heat stress (45°C) treatment. (b) Comparison of heat-induced changes in *F*_q_ in WT and *hsp101* for plants exposed to 45°C for 3 h, 6 h, and 9 h. (d) Comparisons of nonphotochemical quenching (NPQ) of nonstressed WT plants with 3 h, 6 h, and 9 h heat-stressed WT (e) Comparisons of NPQ of 3 h, 6 h, and 9 h heat-stressed WT and *hsp101*. Dashed lines and grey ribbons represent the meanvalue ± 95%confidenceintervals of different plant groups (*n* varies between 17 and 20 for individual groups). The significance of the difference in the size of treated and nontreated WT (a, c) and *hsp101* (b, d) plants each day during the imaging period was determined by Student's *t*-test, with a *p* value below 0.05, 0.01, 0.001, and 0.0001 indicated with ^∗^, ^∗∗^, ^∗∗∗^, and ^∗∗∗∗^, respectively.

As photochemical quenching (*F*_q_) was a unique trait that was associated with differentiation between the WT and heat-sensitive *hsp101* mutant, we examined the heat-induced changes in *F*_q_ throughout the duration of the experiment (Figure 4). The heat exposure resulted in an immediate reduction of *F*_q_ across all heat stress regimes and recovery to the *F*_q_ levels observed for the nontreated plants within 1, 2, or 3 DAS for WT plants exposed to heat stress for 3, 6, and 9 h, respectively (Figure 4(a)). The heat-sensitive *hsp101* mutant showed more severe decrease in *F*_q_ immediately after heat stress exposure in plants exposed to heat stress for 3 and 6 hours (Figure 4(b)). While we also observed a reduction in the nonphotochemical quenching measured at the same light intensity (Lss5) for the WT plants exposed to heat stress (Figure 4(c)), the differences between WT and *hsp101* were less pronounced (Figure 4(d)). The *F*_q_ measured at other light intensities showed similar trends (Figure S6), although the effect of the heat stress on *F*_q_ differed depending on the light intensity at which the chlorophyll fluorescence was studied.

**Figure 5:**
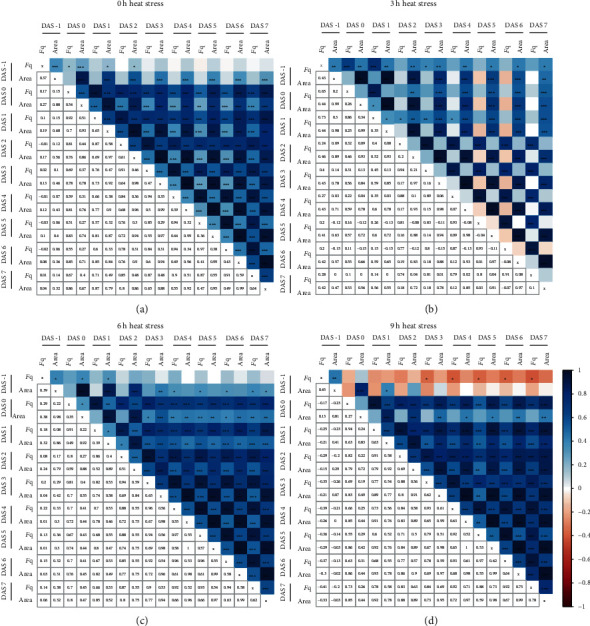
Early changes in photochemical quenching correspond to larger rosette area. The correlation matrix between photochemical quenching (*F*_q_) and rosette area scored at various days after stress (DAS) application for the plants (a) not exposed to heat stress and exposed to (b) 3 h, (c) 6 h, or (d) 9 h of heat stress (45°C). The positive correlation coefficients are indicated with blue, while negative correlation coefficients with red in the upper part of the correlogram. The Pearson correlation coefficient values are listed as numbers in the lower part of each correlogram. The *p* value for each correlation pair, as calculated per *t*-test, are indicated in the upper part of each correlogram with ^∗^, ^∗∗^, ^∗∗∗^, and ^∗∗∗∗^ indicating a *p* value below 0.05, 0.01, 0.001, and 0.0001, respectively.

In order to examine whether the heat stress-induced reduction in *F*_q_ was corresponding to the plant's performance at the later stage of the experiment, we examined the correlations for individual plants between *F*_q_ and rosette area measured at individual DAS (Figure 5). For plants that did not undergo the heat stress treatment, we observed a correlation between *F*_q_ and area across all time points (Figure 5(a)); however, the correlation within the traits (e.g., area at different DAS) was higher than between the traits. Interestingly, for the plants exposed to heat stress for 3 and 6 h, the *F*_q_ measured at 0 and 1 DAS shows strong correlations with the rosette area measured throughout the experiment (Figures 5(b) and 5(c)). This suggests that the maintenance of *F*_q_ could be used as an indicator for plant performance. We also examined the correlations throughout time between other traits that ranked high in logistic regression classification (Table 1) and the rosette area. The heat stress-induced changes in isotropy were negatively correlated with rosette area at earlier time points (DAS 2-4, Figure S7). The rosette compactness at 1 DAS also exhibited a small negative correlation with rosette area at later stages (5-7 DAS) (Figure S8). The slenderness of leaves (SOL) scored at early time points after stress was not correlated with the plant area at 3 or 6 h of heat stress, and at 9 h the rosette area scored at earlier time points was negatively correlated with SOL, suggesting that observed changes in SOL are rather a consequence of reduced rosette area, rather than the cause (Figure S9).

**Figure 6:**
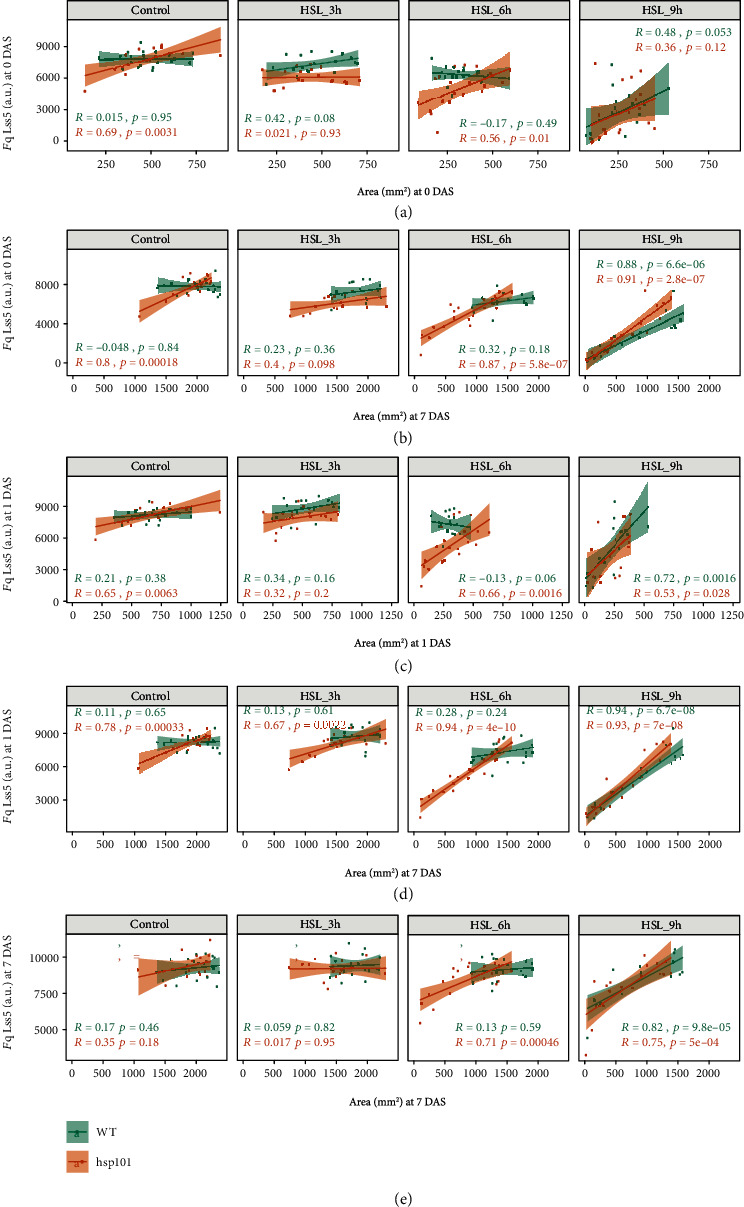
Heat-stress induced reduction in photochemical quenching indicates heat susceptibility. The correlation between photochemical quenching (*F*_q_) and rosette area was examined for plants not exposed to heat stress and plants exposed to 3 h, 6 h, or 9 h of heat stress (45°C). The correlation was examined between *F*_q_ scored at 0 days after stress (DAS) imposition and rosette area at (a) 0 DAS and (b) 7 DAS and between *F*_q_ scored at 1 DAS and rosette area at (c) 1 DAS and (d) 7 DAS, as well as (e) *F*_q_ scored at 7 DAS and rosette area at 7 DAS. The correlation coefficients (*R*) for individual genotypes (WT and *hsp101*) as well as the corresponding *p* values as calculated per Pearson's correlation *t*-test are indicated with different colours. The lines in each graph represent the regression line for each treatment and genotype combination. The individual plants are represented by individual dots.

As correlations between the rosette area and compactness or isotropy were only weakly significant (0.01 < *p* value < 0.05), and the trends were only observed in plants exposed to heat stress for 3 h, the correlation between early changes in *F*_q_ and rosette area size suggests that the maintenance of *F*_q_ might be causal to plant performance at a later stage. To examine these correlations in more details, the *F*_q_ at 0 and 1 DAS was plotted for individual heat stress regime and the correlation between rosette area in individual genotypes was examined (Figure 6). The correlation between *F*_q_ and area when both traits are scored at 0 DAS is weak and nonsignificant in most cases (Figure 6(a)). However, when we examined the correlation between *F*_q_ scored at 0 DAS with rosette area at later time points, the correlation coefficients increased for the heat stress-treated samples (Figure 6(b) and Figure S10). Interestingly, across the increasing heat stress exposure, the correlations between *F*_q_ at DAS 0 and rosette area at later DAS increased more decidedly for the heat-susceptible *hsp101*, suggesting that the maintenance of photochemical quenching had even higher importance for this heat-susceptible genotype. Similar correlations were for *F*_q_ measured at 1 DAS, where the correlations between *F*_q_ and rosette area measured both at 1 DAS were relatively weak (Figure 6(c)) and increase when rosette area is measured at later time points (Figure 6(d) and Figure S11). The *F*_q_ measured at 7 DAS also shows significant correlations with the rosette area measured at the same time (Figure 6(e)); however, the correlation coefficients are lower and the *p* values are higher than for the correlations between early changes in *F*_q_ and later size of the rosette area. These results are strongly suggestive that photochemical quenching might be an important component of heat stress tolerance and that maintenance of *F*_q_ is particularly important in the heat-susceptible lines.

## 4. Discussion

Increasing temperature is one of the most important environmental factors affecting agricultural productivity worldwide. Improving our understanding of the mechanisms underlying plant heat stress responses will facilitate the development of technologies and breeding strategies for improving plant thermotolerance. While previous studies identified a number of traits which are important indicators of heat and drought tolerance during flowering stage (Chen et al., 2019), the scoring of these traits requires specialized equipment and requires plants to reach the reproductive stage. Developing phenotypic indicators of heat stress tolerance which can be scored at the vegetative stage would accelerate the selection of germplasm. In this manuscript, we show an example of how high-throughput phenotyping can be used to screen for heat tolerance-related traits, providing more insight in the physiological processes contributing to thermotolerance.

By studying the heat-induced changes in plant size, morphology, temperature, and chlorophyll fluorescence, we identified a set of phenotypes like leaf temperature, maximum quantum yield, and slenderness of leaves that show immediate response to heat stress. By evaluating three different heat stress regimes (3, 6, and 9 h at 45°C, Figure 1), we identified which trends were observed across all treatments. By including a heat-sensitive *hsp101* mutant, we were able to distinguish which phenotypes were informative in distinguishing between the WT and heat-sensitive lines (Table 1). We observed that *hsp101* plants showed a more severe decrease in plant growth and quantum yield compared to the WT plants and that the difference between genotypes was most evident across the plants exposed to heat stress for 6 h (Figure 2). The rapid change in quantum yield seems to be specific to heat stress, as Arabidopsis plants exposed to salt stress did not show reduced photosynthetic yield with similar length of the experiment [18], while drought decreased quantum yield only at the later stage of stress exposure [17]. While the changes in quantum yield and photochemical quenching were observed immediately after the heat stress, the heat stress also affected rosette morphology at later time points, including the reduction in slenderness of leaves, compactness, and increased rosette isotropy (Figure S2). Such changes in rosette morphology were less apparent under salt stress [18]. These differences between heat, drought, and salt reflect the nature of abiotic stress, with heat stress exposure being the most acute, while the severity of salt and drought stress increases gradually over long periods of time. The observed differences highlight the importance of selecting the physiologically relevant levels of stress, considering the differences in the nature of individual abiotic stress that the plants are exposed to in the environment. Summarizing, results presented in this study showed that plant physiological responses to high temperature are complex and temporal in their nature, with short-term changes being captured best with chlorophyll fluorescence (*F*_m_, *F*_0_ and QY max, *F*_q_) and leaf temperature, while heat-induced changes in rosette morphology (rosette area, perimeter, compactness, and slenderness of leaves) are observed at an extended time after application of heat stress. While the majority of the heat stress studies focus on survival, we think that using the combination of these parameters and screening them throughout time will provide a better understanding of processes underlying heat tolerance. Future studies using a higher number of genotypes and plant species will help to elucidate the genotype-specific effect of heat stress responses and provide consensus traits contributing to heat stress tolerance across multiple genetic backgrounds.

In this study, we observed that while heat stress exposure increased the leaf angle in WT, this response was absent in *hsp101* mutant lines (Figure S3). While the response is obvious to the human eye, it proved difficult to detect it from the available morphology parameters, which use only topview images. The increased leaf angle was earlier suggested to have an adaptive advantage under high temperature, increasing the cooling capacity of the plant [30, 34]. As we did not observe any significant differences in leaf temperature between the WT and *hsp101* mutants (Figure S5), the cooling advantage of this response is yet to be demonstrated. Nevertheless, the *hsp101* showed a greater reduction in maximum quantum yield compared to WT (Figure 2) and a lower decrease in photochemical and nonphotochemical quenching (Figure 4). However, as the change in leaf angle was only observed at 1 DAS, it is unlikely that this response to cause the decreased photosynthetic efficiency. How the increase in leaf angle and photosynthetic quenching mechanisms are orchestrated by hsp101 is beyond the scope of this study. If the leaf angle is to be used for future assays, it is our suggestion to include the 3D measurements of the rosettes using the 3D scanner technology and/or sideview image.

While high-throughput phenotyping provides more information on plant performance using the nondestructive measurements, the number of the collected direct and derived measurements (Table S1) can be overwhelming for effective trait evaluation. Machine learning algorithms can help navigate through the complex dataset. The previous study used a combination of random forest and support vector machine models to identify most distinguishing root traits and cultivar differentiation across European pea cultivars [35]. In our study, we used logistic regression to identify the most informative traits which would enable the differentiation between the WT and heat-sensitive *hsp101* mutant (Table 1). Logistic regression models are suitable only for bivariate analyses, differentiating between tolerant versus susceptible genotypes. For future studies where more genotypes would be screened, we advise using other machine learning models, such as decision trees or multiple regression models to access the quantitative nature of heat stress tolerance. The logistic regression model results supported our earlier analysis that the 6 h heat treatment is most informative for differentiating between WT and heat-sensitive genotype and highlighted the significance of morphology traits, direct chlorophyll fluorescence measurements, and photochemical quenching (Table 1). These results lead us to investigate changes in photochemical quenching in more detail (Figure 4). We observed that the maintenance of photochemical quenching was positively correlated with larger rosettes at later time points (Figure 5) and that this correlation was predominantly found in the plants exposed to heat stress (Figure 6). The contributions of nonphotochemical quenching to heat stress tolerance [36], as well as other abiotic stress [37], are widely described in earlier literature. The photochemical quenching is related to the redox state of the first electron acceptor of photosystem II [38], but how this process is affected by stress and what is its contribution to overall environmental stress tolerance remains unknown. While we want to stress that the correlation does not prove causation, the observed correlation between photochemical quenching and thermotolerance will be an important cornerstone in future research of heat stress responses.

Using kinetic chlorophyll fluorescence assays, such as light-curve protocol [24, 25] used in this study, provided new insights in dynamic changes to photosynthetic efficiency and heat stress-induced photochemical quenching. We think that the new phenotypic traits, presented in this manuscript, will provide better insight and identify novel players contributing to overall plant performance and heat stress tolerance. As processes contributing to overall thermotolerance are complex [39] and can be acquired in various ways [40], the assays focusing on seedling survival or hypocotyl elongation are of limited value. Using more refined phenotypes, such as rosette morphology parameters or traits derived from direct chlorophyll fluorescence, measurements will in future studies contribute to the discovery of genes and processes which are small and transient, but a significant contribution to thermotolerance.

Summarizing, we identified a series of phenotypes which correspond to increased heat stress sensitivity of the hsp101 mutant line, including QY max, rosette size, and rosette compactness. Our logistic regression model identified that early changes in *F*_q_ can serve as an early indicator of plant performance at the later stage. The traits reported in this manuscript can be measured throughout time using image-based phenotyping, making the discussed traits suitable for high-throughput screening of germplasm. Future studies will reveal the genotype and developmental stage specificity of the contribution of reported traits to overall heat stress tolerance.

## Figures and Tables

**Table 1:** Logistic regression classification accuracies to determine the WT and *hsp101* plants under different treatments.

Treatment	Control	3 h HS	6 h HS	9 h HS
Accuracy (subset of traits)	61.1%	68.2%	65.2%	58.4%
Accuracy (all traits)	67.9%	79.0%	81.5%	65.7%
Top 10 traits	Temperature	RMS	Compactness	RMS
	SOL	Isotropy	Isotropy	Temperature
	*F*_t_′ Lss2	SOL	Temperature	SOL
	*F*_t_′ Lss4	*F*_0_′ Lss2	SOL	*F*_t_′ Lss5
	*F*_t_′ Lss3	*F*_0_	Perimeter	*F*_0_′ Lss5
	*F*_t_′ Lss6	*F*_t_′ Lss5	*F*_q_′ Lss5	*F*_0_
	*F*_q_′ Lss3	*F*_q_′ Lss5	*F*_t_′ Lss4	*F*_t_′ Lss1
	*F*_q_′ Lss6	*F*_0_′ Lss3	*F*_t_′ Lss3	*F*_t_′ Lss6
	*F*_0_′ Lss3	*F*_t_′ Lss4	*F*_v_′ Lss4	*F*_t_′ Lss2
	*F*_q_′ Lss2	*F*_t_′ Lss3	*F*_t_′ Lss2	*F*_q_′ Lss6

SOL: slenderness of leaves; RMS: rotational mass symmetry; *F*_0_: minimal fluorescence in the dark-adapted state; *F*_0_′: minimal fluorescence in the light-adapted state; *F*_t_′: steady-state fluorescence in the light-adapted state; *F*_q_: photochemical quenching. Lss indicates the light intensity at which individual chlorophyll fluorescence traits were determined (see Materials and Methods).

## Supplementary Materials

Supplementary 1 Table S1: list of direct and indirect chlorophyll fluorescence and morphological parameters used in this study. Table S2: the linear model testing the effect of treatment, genotype, and interaction between genotype and treatment for 3 h, 6 h, and 9 h treatments for individual phenotypic traits.

Supplementary 2 Figure S1 WT and hsp101 plants are indistinguishable when not exposed to heat stress.

Supplementary 3 Figure S2 Characterization of heat-induced morphological responses in WT and hsp101 after 6 h heat treatment.

Supplementary 4 Figure S3 Heat stress-induced changes to minimum chlorophyll fluorescence.

Supplementary 5 Figure S4 Heat stress-induced changes to maximum chlorophyll fluorescence.

Supplementary 6 Figure S5 Heat stress-induced changes to leaf temperature.

Supplementary 7 Figure S6 Characterization of heat-induced changes in photochemical quenching in WT and hsp101.

Supplementary 8 Figure S7 Temporal correlation between heat stress-induced changes in rosette isotropy and rosette area.

Supplementary 9 Figure S8 Temporal correlation between heat stress-induced changes in rosette compactness and rosette area.

Supplementary 10 Figure S9 Temporal correlation between heat stress-induced changes in slenderness of leaves and rosette area.

Supplementary 11 Figure S10 Heat stress-induced reduction in *F*_q_ at 0 DAS indicates heat susceptibility.

Supplementary 12 Figure S11 Heat-stress induced reduction in *F*_q_ at 1 DAS indicates heat susceptibility.
