# Supplementary material for: The Use of High-Throughput Phenotyping for Assessment of Heat Stress-Induced Changes in Arabidopsis
Source: Plant Phenomics. 2020 Jul 17;2020:3723916. doi: 10.34133/2020/3723916 (PMC7706305; doi:10.34133/2020/3723916)
Supplement: Supplementary 1 — Table S1: list of direct and indirect chlorophyll fluorescence and morphological parameters used in this study. Table S2: the linear model testing the effect of treatment, genotype, and interaction between genotype and treatment for 3 h, 6 h, and 9 h treatments for individual phenotypic traits. [file 3723916.f1.zip › 3723916.f1/Table S1.docx]

**Table S1: List of direct and indirect chlorophyll fluorescence and morphological parameters used in this study.** Q_A_ : Quinone A, the primary stable electron acceptor of PSII centres, PSII: Photosystem II, NPQ: non-photochemical quenching

| **Symbol** | **Formula** | **Name** | **Description** |
| --- | --- | --- | --- |
| F0 | Measured | minimum fluorescence in dark-adapted state | QA oxidized (qP=1), non-photochemical quenching relaxed (NPQ=0) |
| Fm | Measured | maximum fluorescence in dark-adapted state | QA reduced (qP=0), non-photochemical quenching relaxed (NPQ=0) |
| Fv | F_m_- F_o_ | variable fluorescence in dark-adapted state | variable fluorescence increment that is due the transition from dark-adapted state with all-open reaction centers to the all-closed state during saturating flash of light |
| QY_max | (F_m_- F_o_) / F_m_ | maximum PSII quantum yield | maximum PSII quantum yield in dark-adapted state |
| F_m_’ | Measured | steady-state maximum fluorescence in light | level of fluorescence when Q_A_ is maximally reduced (PSII centers closed) |
| Ft | Measured | steady-state fluorescence in light | steady-state fluorescence level that results from a dynamic equilibrium of plastoquinone reducing and re-oxidizing processes and from non-photochemical quenching |
| F_o_’ | Measured | minimum fluorescence in light-adapted state | level of fluorescence when Q_A_ is maximally oxidized (PSII centers open) |
| F_v_’ | F_m_’- F_o_’ | variable fluorescence in light-adapted state | the ability of PSII to perform photochemistry (Q_A_ reduction) |
| F_q_ | F_m_’- F_t_’ | difference between F_m_ and F_t_ in light-adapted state | photochemical quenching of fluorescence by open PSII center |
| QY’ | (F_m_’- F_t_’) / F_m_’ | steady-state PSII quantum yield in light | actual quantum yield of PSII photochemistry for a light-adapted state |
| F_v_’/F_m_’ | (F_m_’-F_o_’) /F_m_’ | PSII quantum yield of light-adapted sample in steady-state | maximal quantum yield of PSII photochemistry for light-adapted state |
| NPQ | (F_m_-F_m_’) /F_m_’ | steady-state non-photochemical quenching | steady-state non-photochemical quenching |
| qP’ | (F_m_’- F_t_’) / (F_m_’- F_o_’) | coefficient of photochemical quenching in steady-state | coefficient of photochemical quenching which estimates fraction of the open PSII reaction centers |
| qN’ | (F_m_- F_m_’) / (F_m_’- F_0_’) | coefficient of non-photochemical quenching in steady state | coefficient of non-photochemical quenching which estimates fraction of variable chlorophyll fluorescence quenched by non-photochemical processes |
| Area | Measured | Rosette area | total area covered with plant |
| Perimeter | Measured | Rosette perimeter | length of the plant perimeter |
| Roundness | ratio | roundness | ratio between area and perimeter or its convex hull |
| Compactness | ratio | compactness | ratio between area and surface of convex hull |
| RMS | ratio | rotational mass symmetry | difference between convex hull area and circle which has center in plant centroid and radius proportional to area weighted by compactness |
| Eccentricity | ratio | eccentricity | ratio between the distance of the foci of the ellipse with same variance as a plant and its major axis length |
| SOL | ratio | slenderness of leaves | ratio between squared sum of leaf lengths and area |
| Isotropy | Measured | isotropy | uniformity in all orientations |
