# Supplementary figures and images for: The Use of High-Throughput Phenotyping for Assessment of Heat Stress-Induced Changes in Arabidopsis
Source: Plant Phenomics. 2020 Jul 17;2020:3723916. doi: 10.34133/2020/3723916 (PMC7706305; doi:10.34133/2020/3723916)

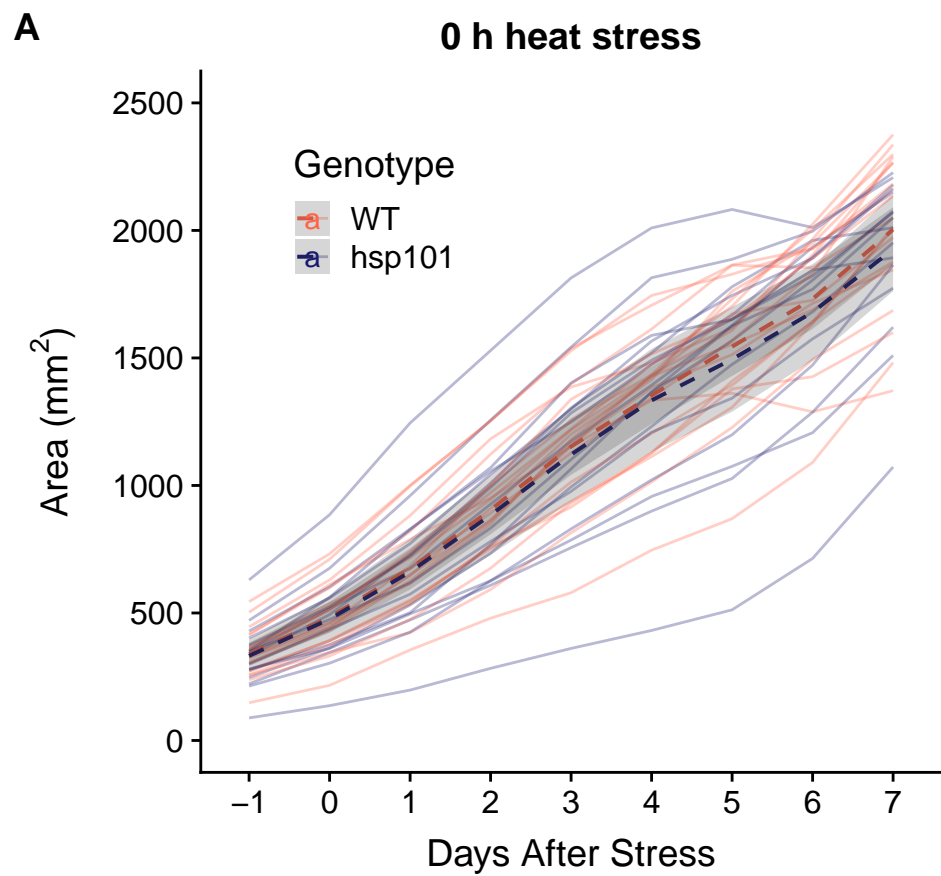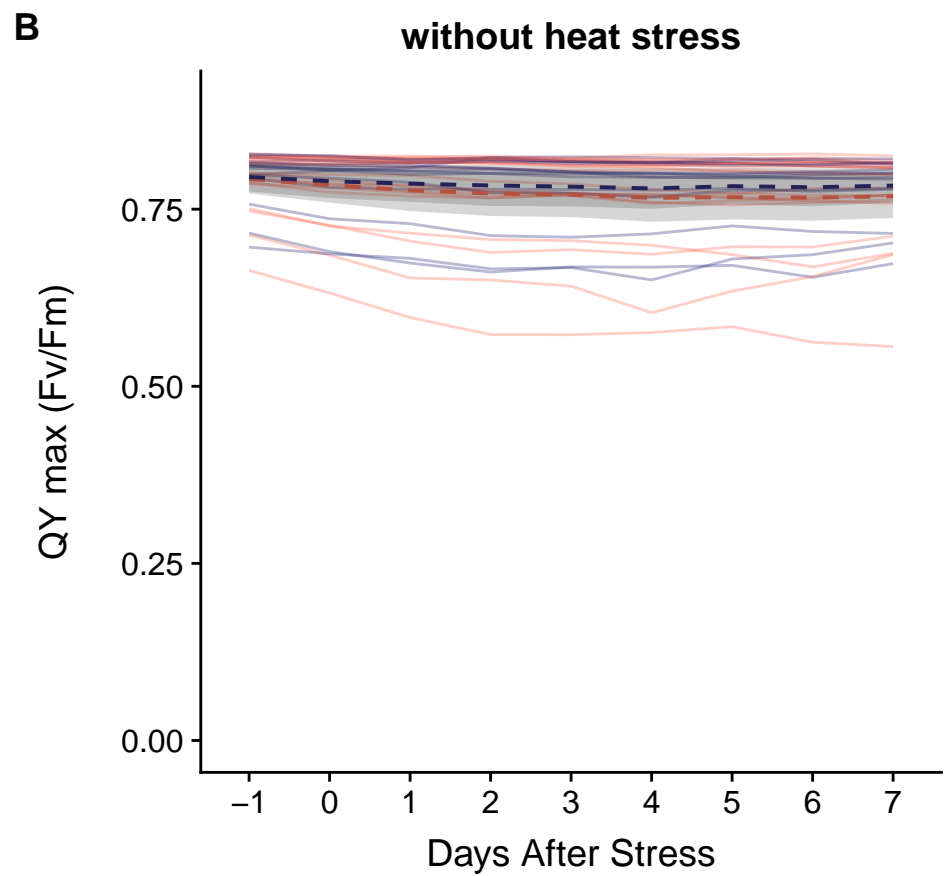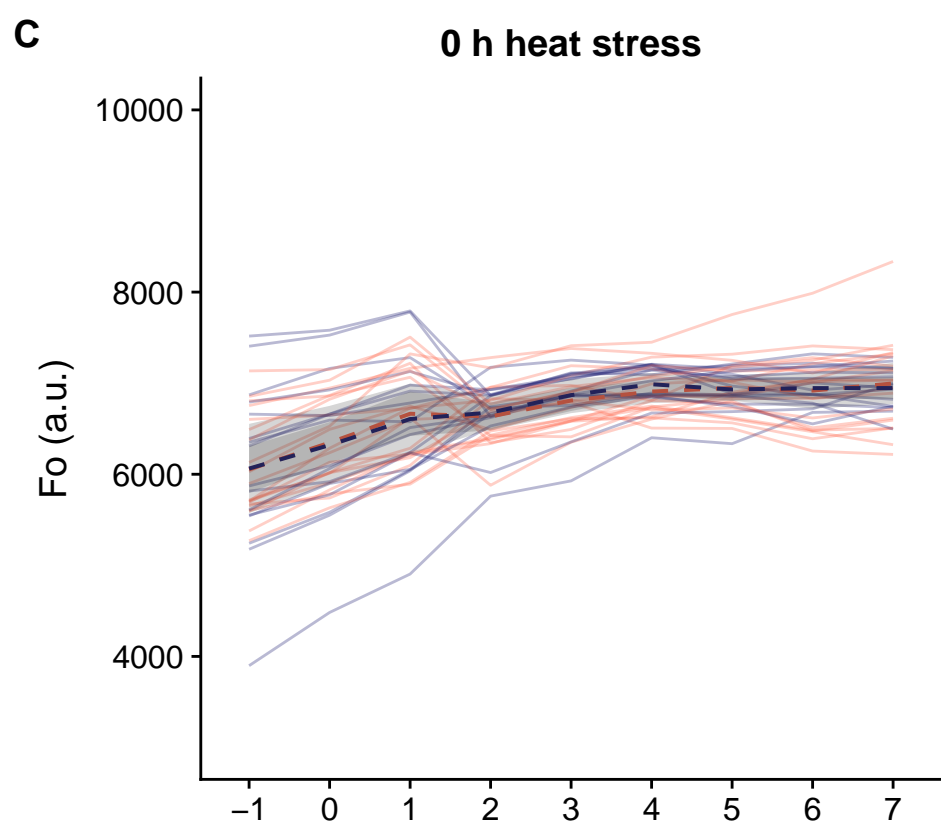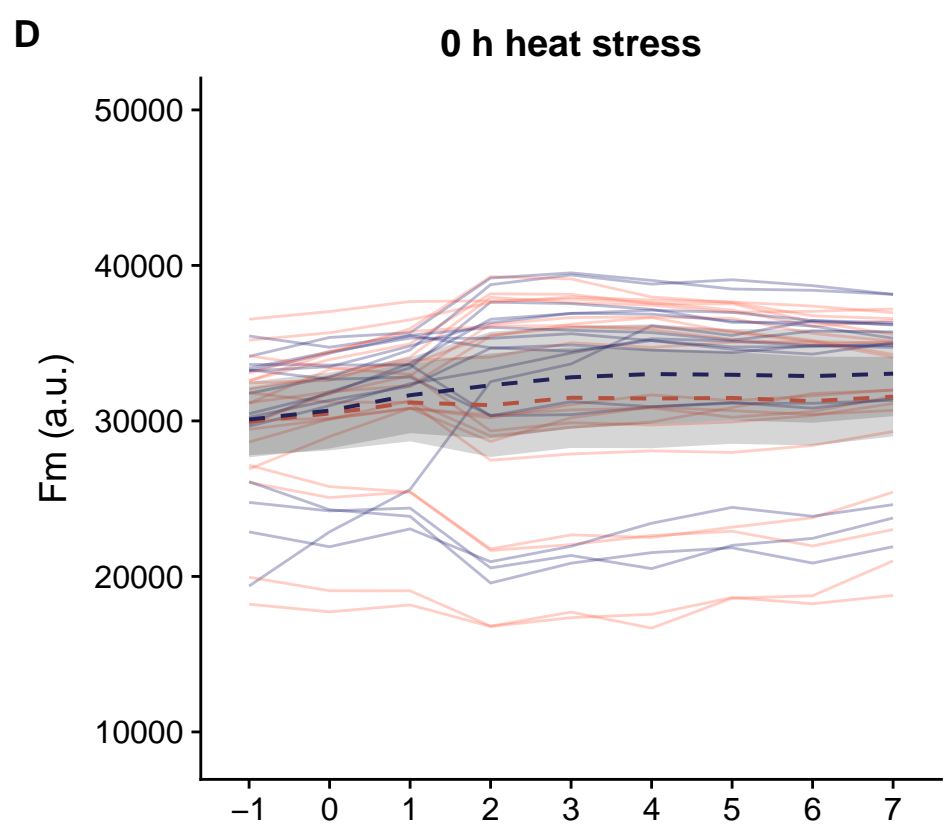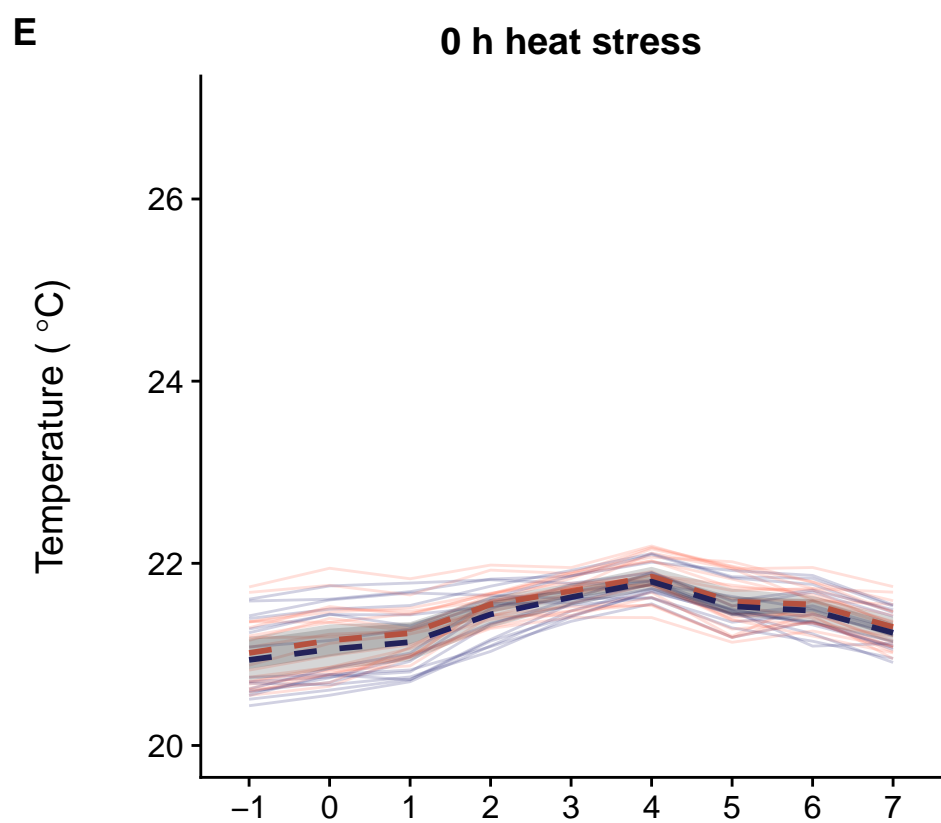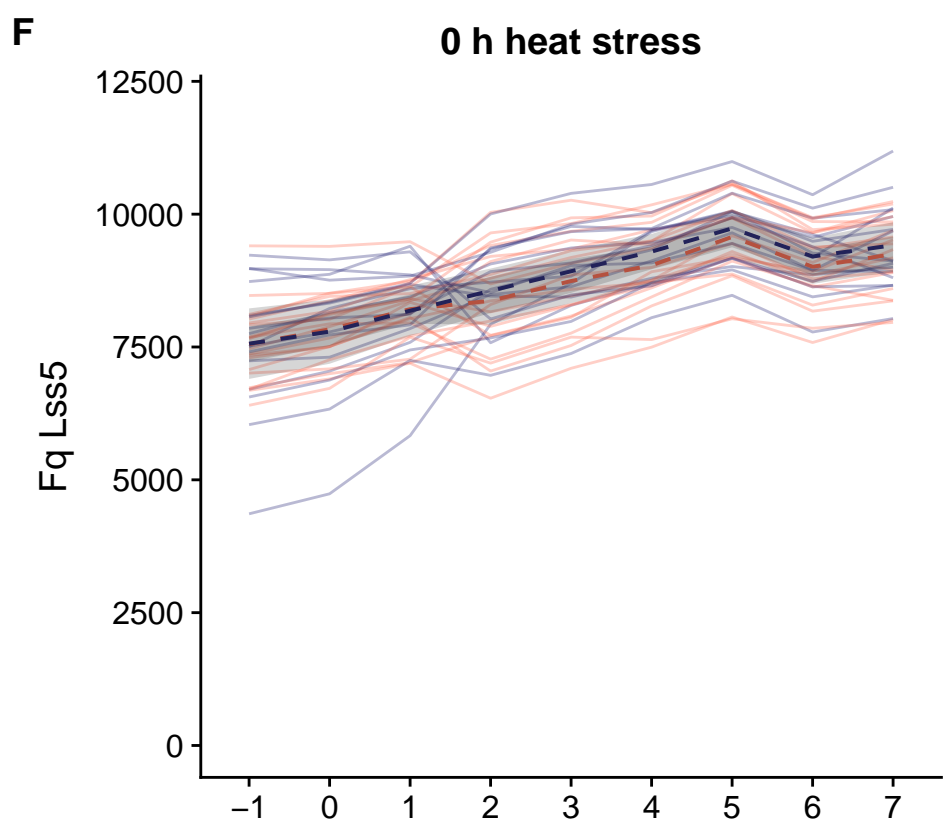

Supplement: Supplementary 2 — Figure S1 WT and hsp101 plants are indistinguishable when not exposed to heat stress. [file 3723916.f2.pdf]

A

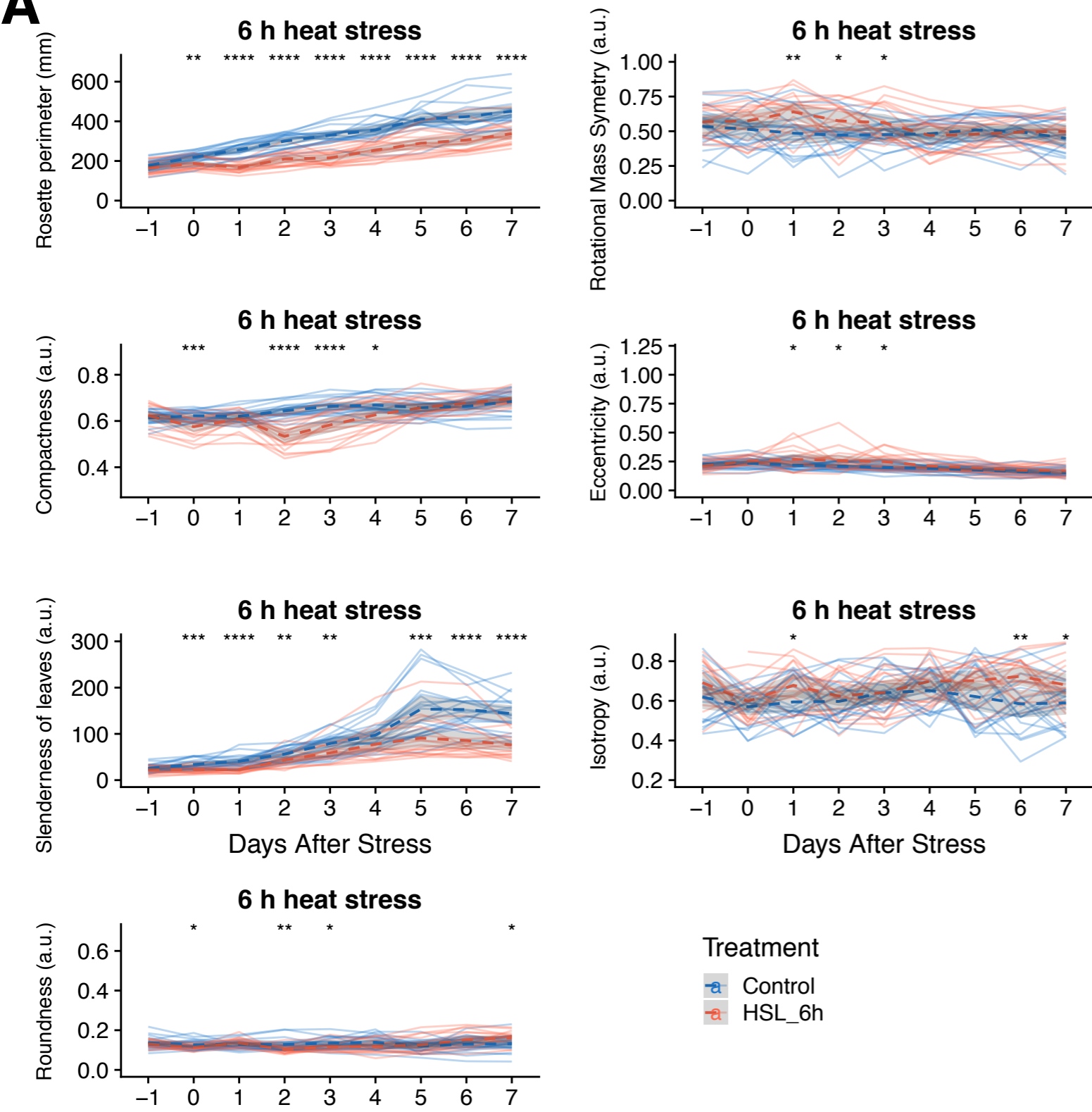

B

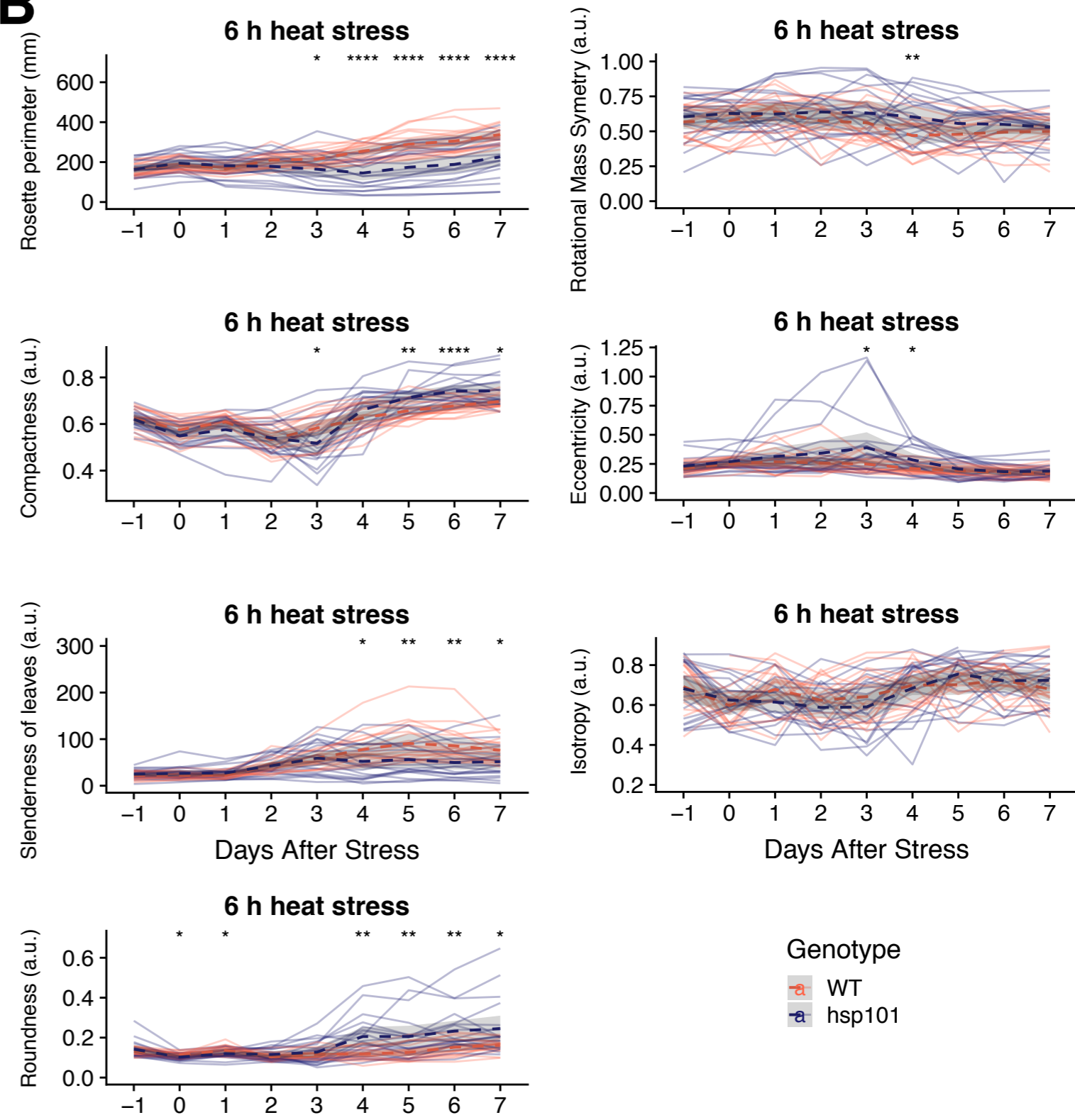

C

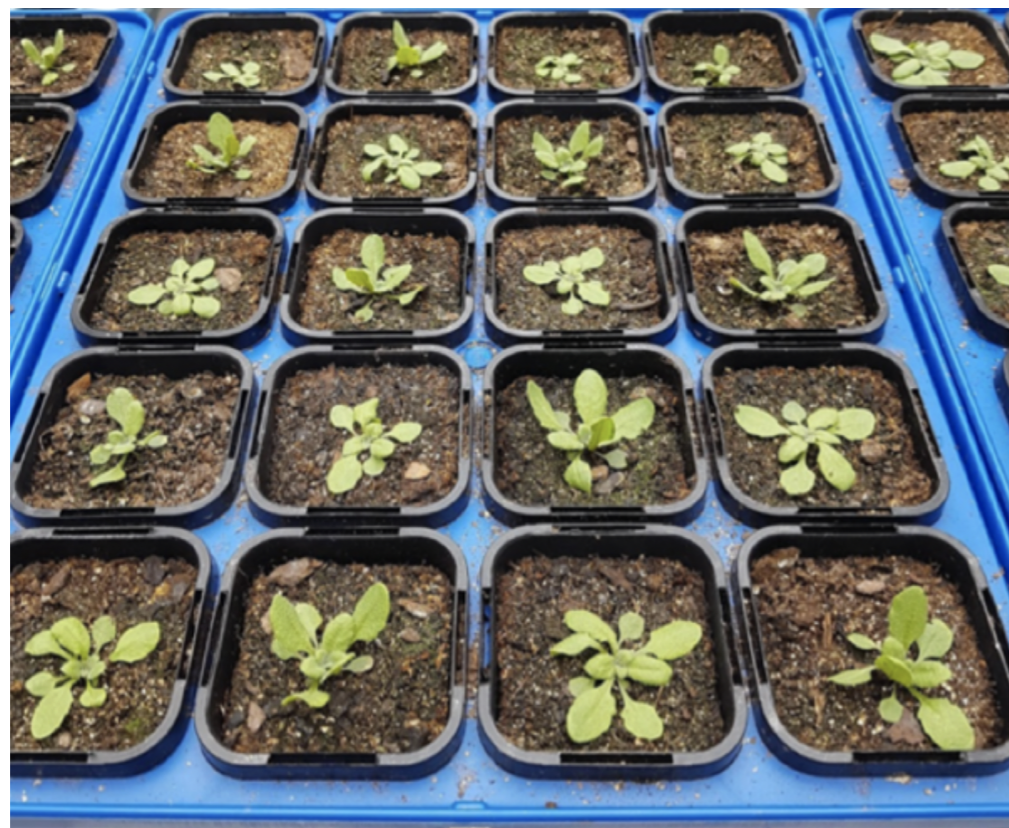

|               |               |               |               |
|---------------|---------------|---------------|---------------|
| <i>hsp101</i> | WT            | <i>hsp101</i> | WT            |
| WT            | <i>hsp101</i> | WT            | <i>hsp101</i> |
| <i>hsp101</i> | WT            | <i>hsp101</i> | WT            |
| WT            | <i>hsp101</i> | WT            | <i>hsp101</i> |
| <i>hsp101</i> | WT            | <i>hsp101</i> | WT            |

Supplement: Supplementary 3 — Figure S2 Characterization of heat-induced morphological responses in WT and hsp101 after 6 h heat treatment. [file 3723916.f3.pdf]

**A****3 h heat stress**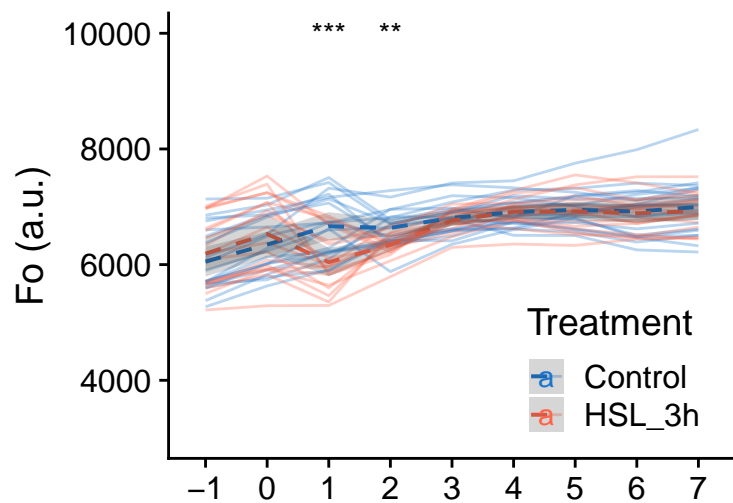**B****3 h heat stress**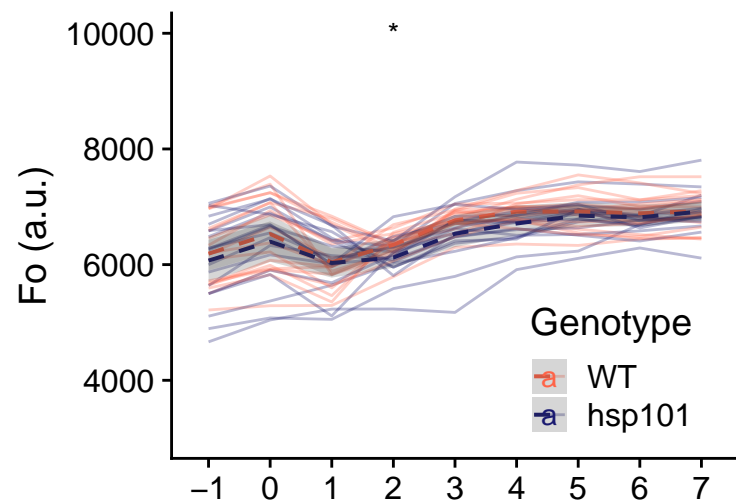**6 h heat stress**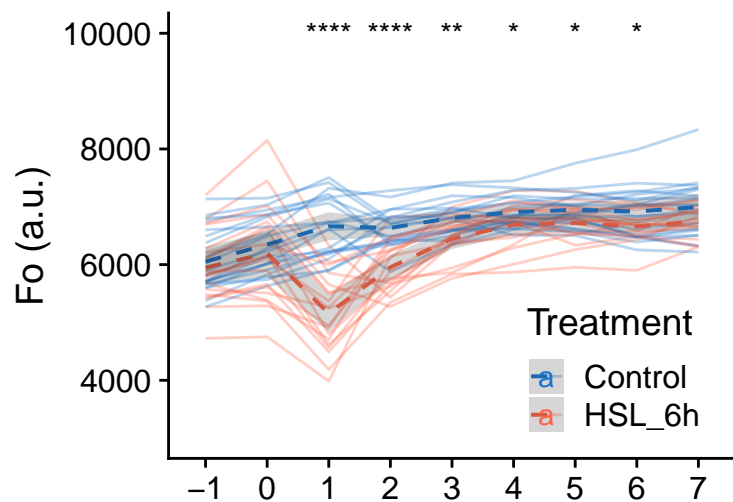**6 h heat stress**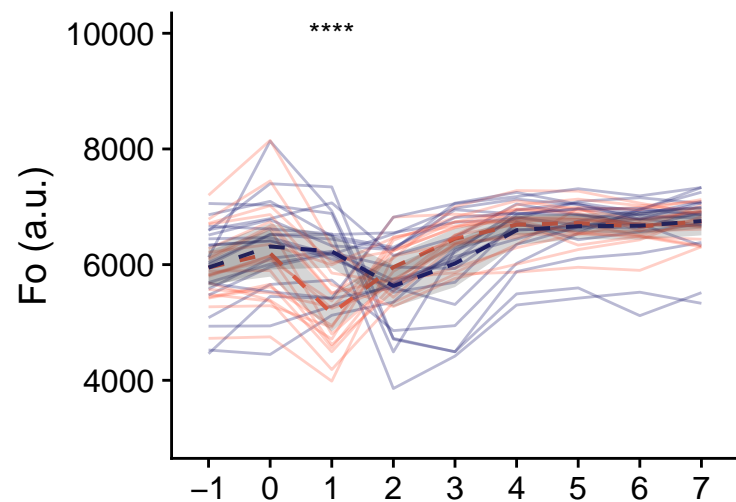**9 h heat stress**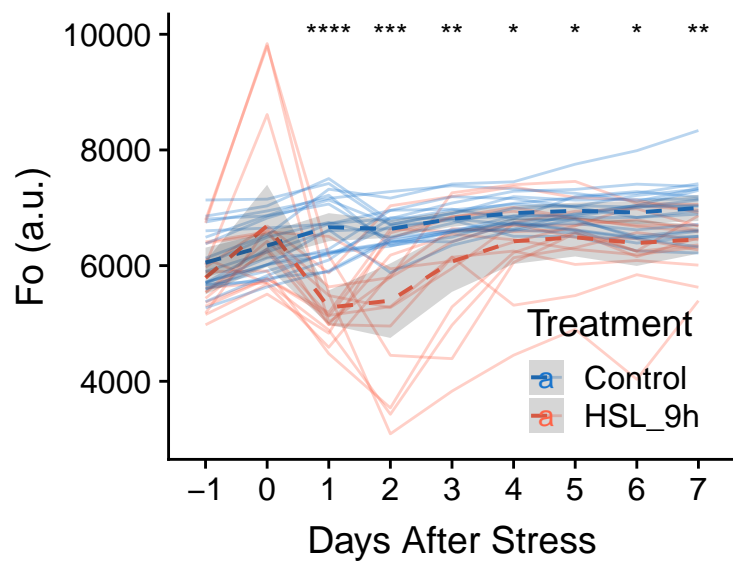**9 h heat stress**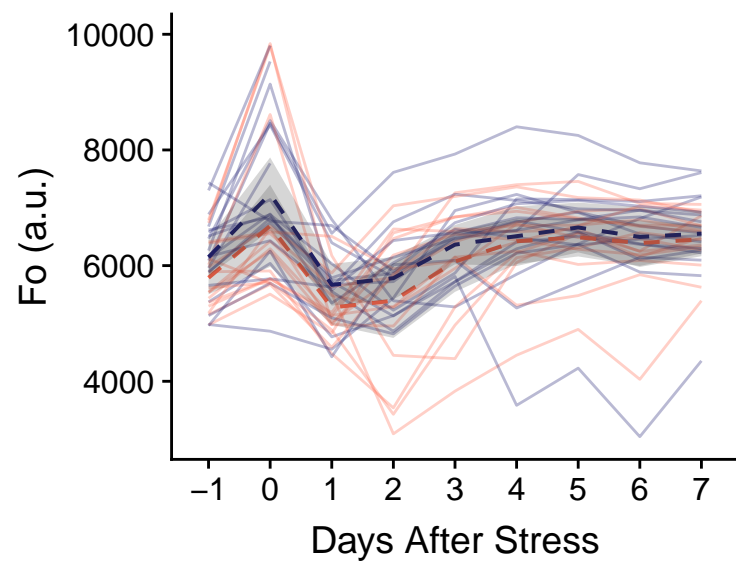

Supplement: Supplementary 4 — Figure S3 Heat stress-induced changes to minimum chlorophyll fluorescence. [file 3723916.f4.pdf]

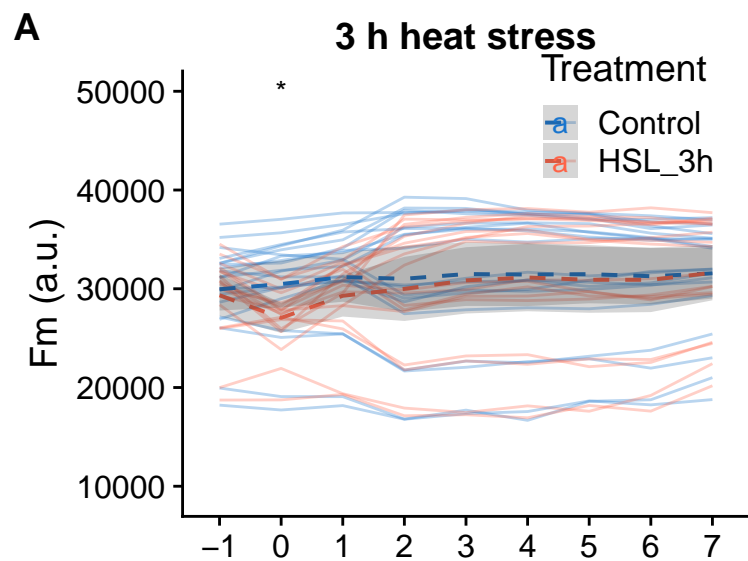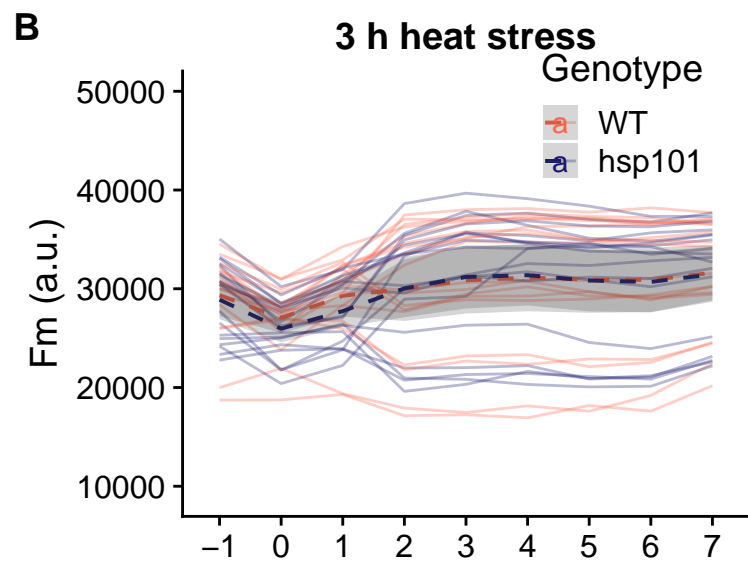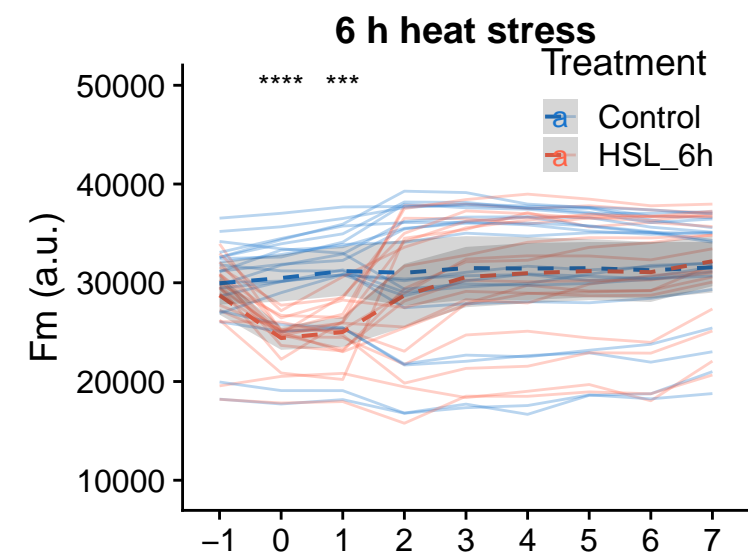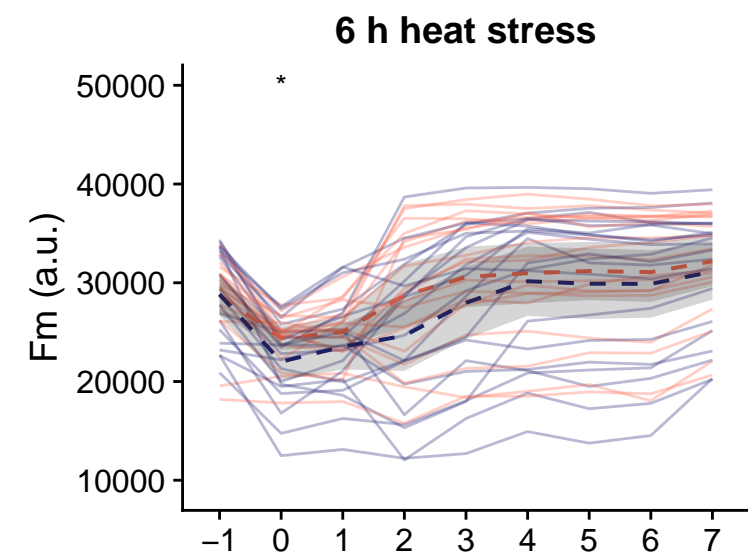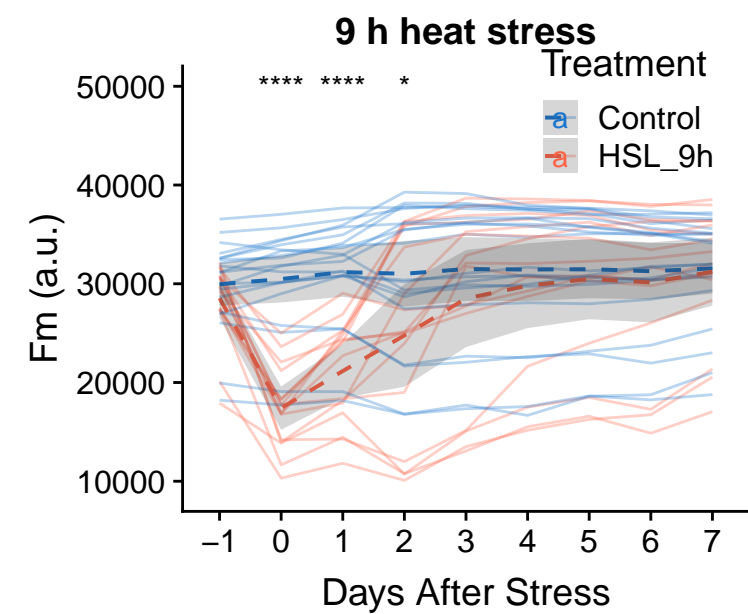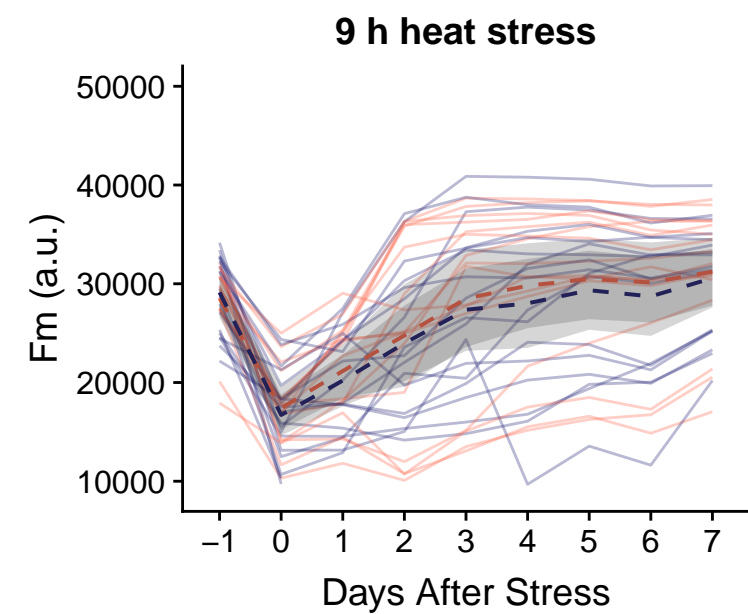

Supplement: Supplementary 5 — Figure S4 Heat stress-induced changes to maximum chlorophyll fluorescence. [file 3723916.f5.pdf]

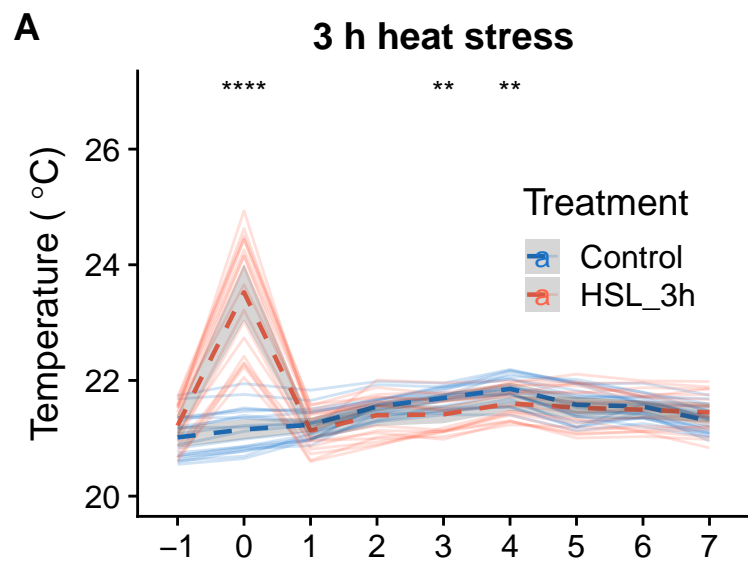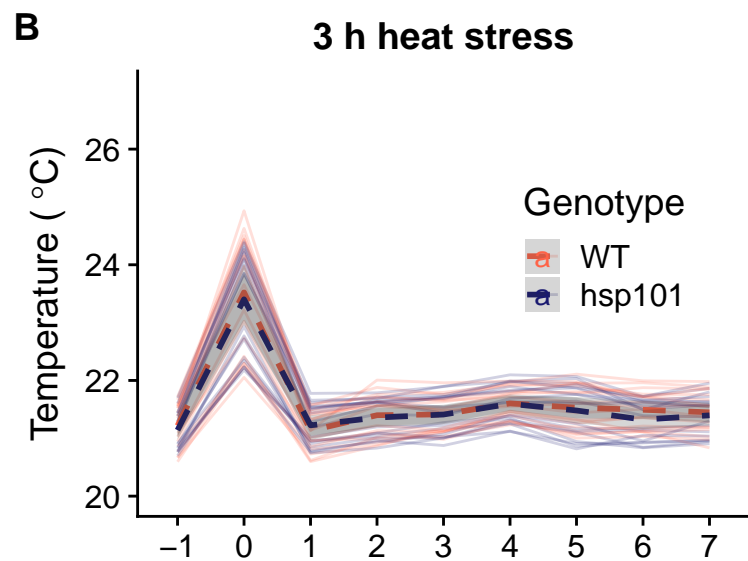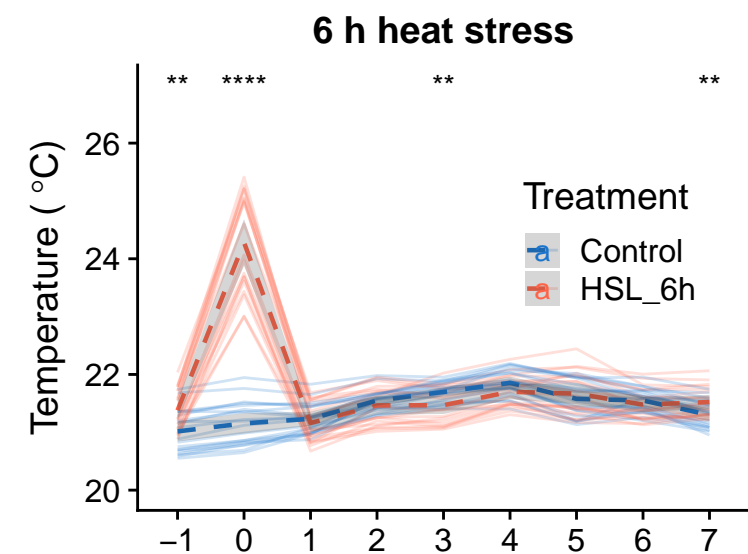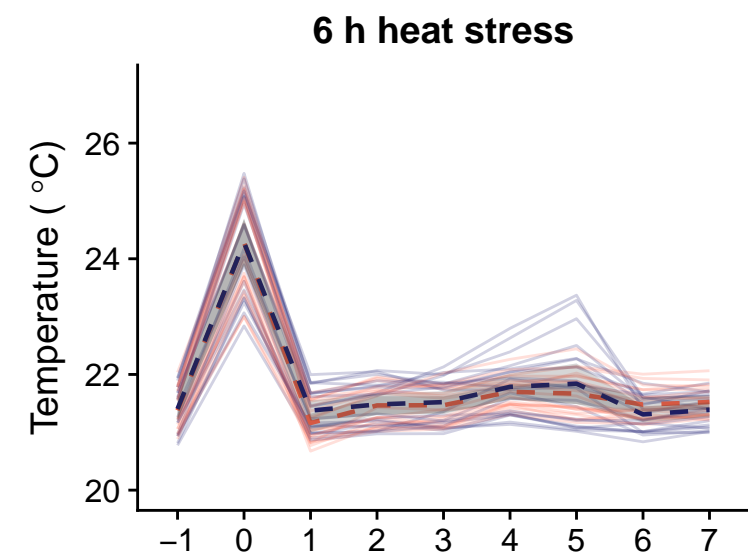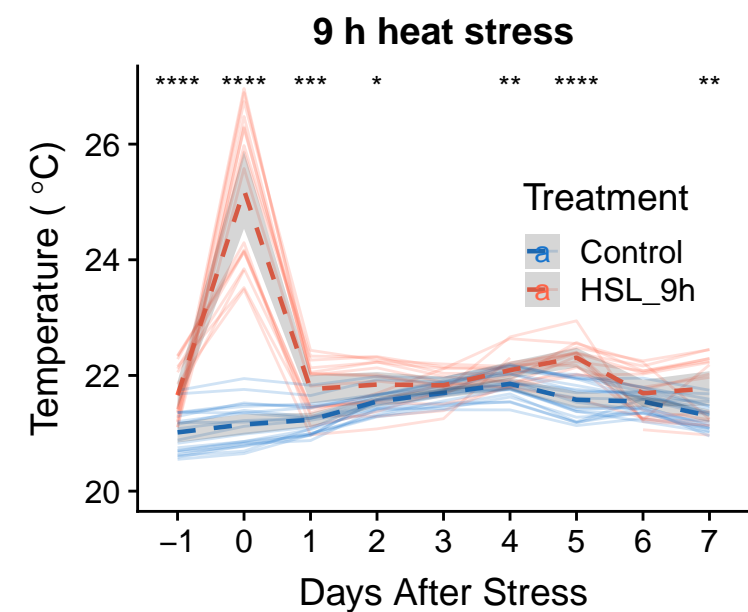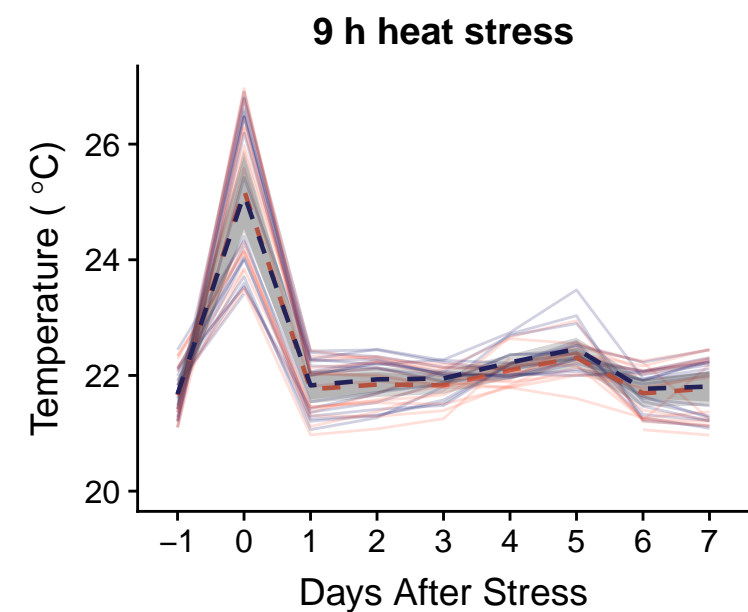

Supplement: Supplementary 6 — Figure S5 Heat stress-induced changes to leaf temperature. [file 3723916.f6.pdf]

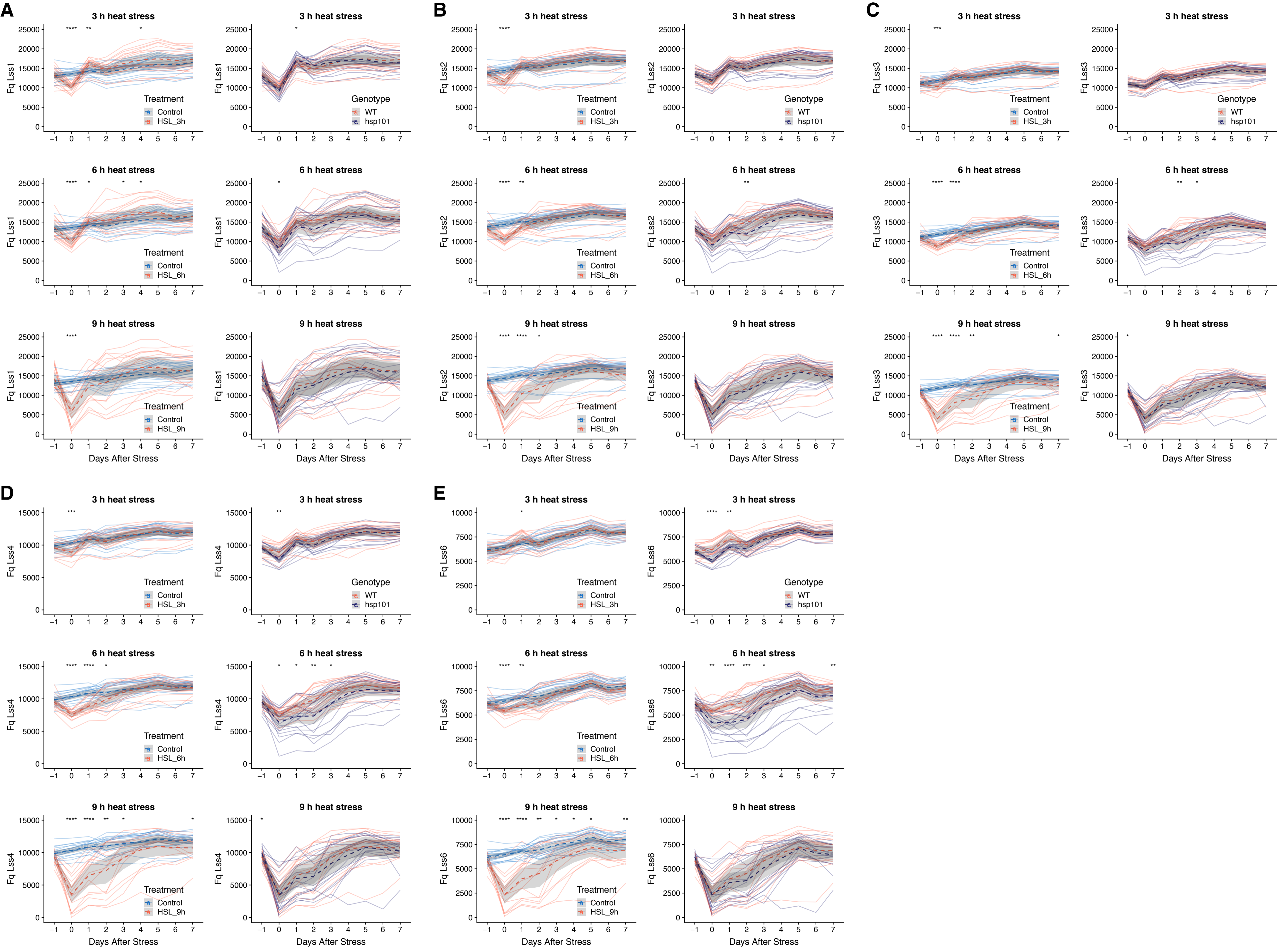

Supplement: Supplementary 7 — Figure S6 Characterization of heat-induced changes in photochemical quenching in WT and hsp101. [file 3723916.f7.pdf]

# A0 h heat stress

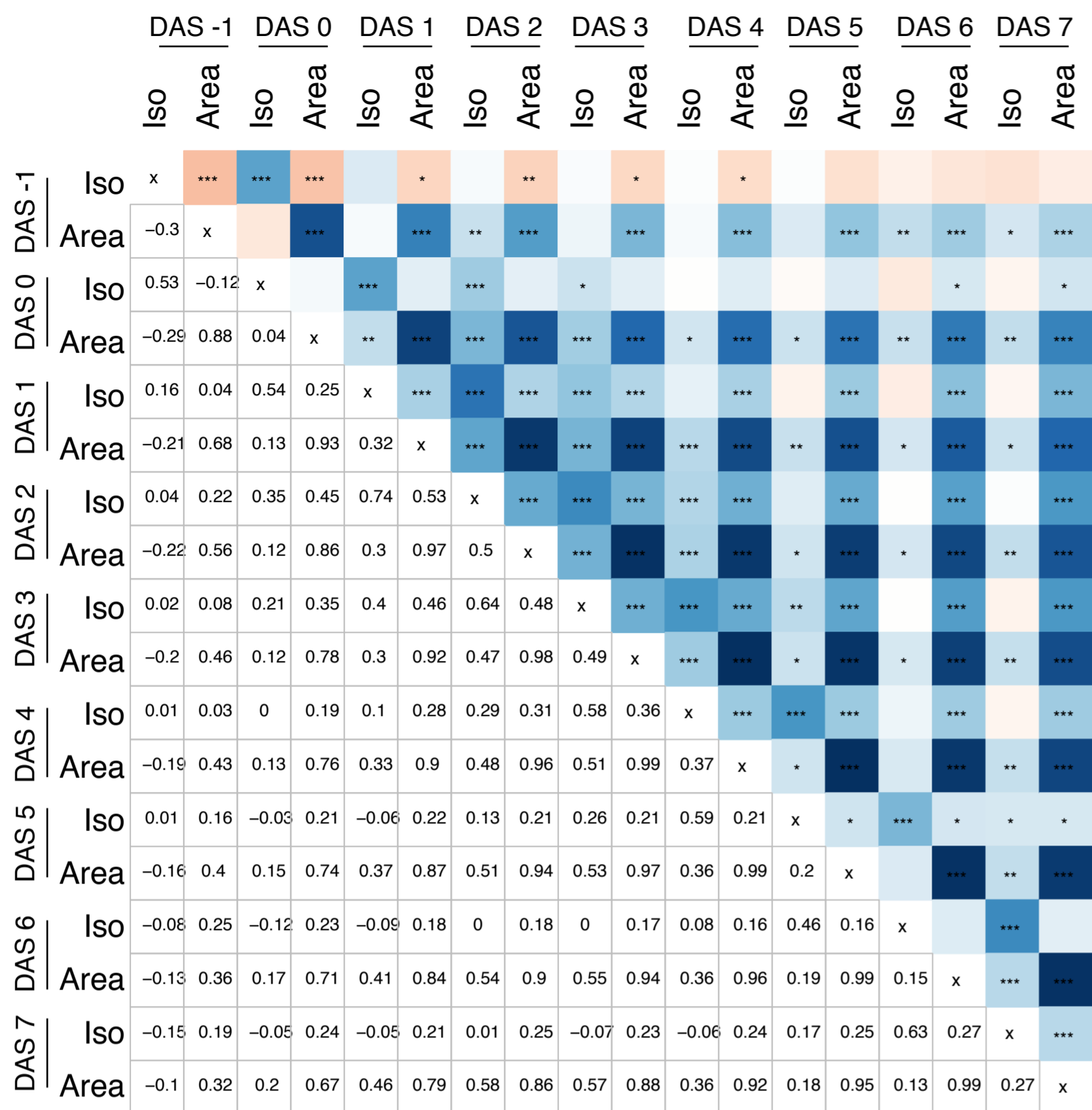

# B3 h heat stress

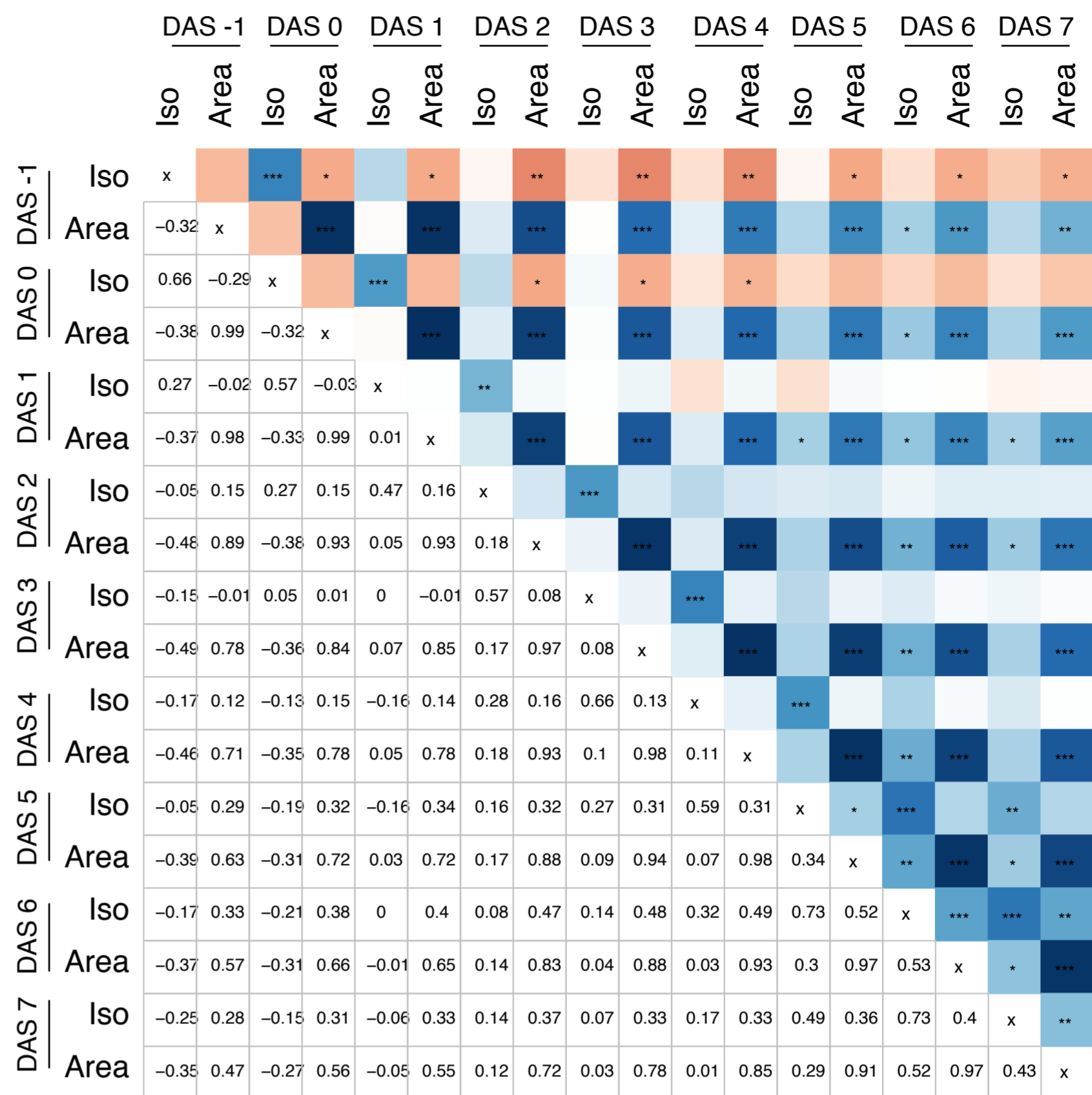

# C6 h heat stress

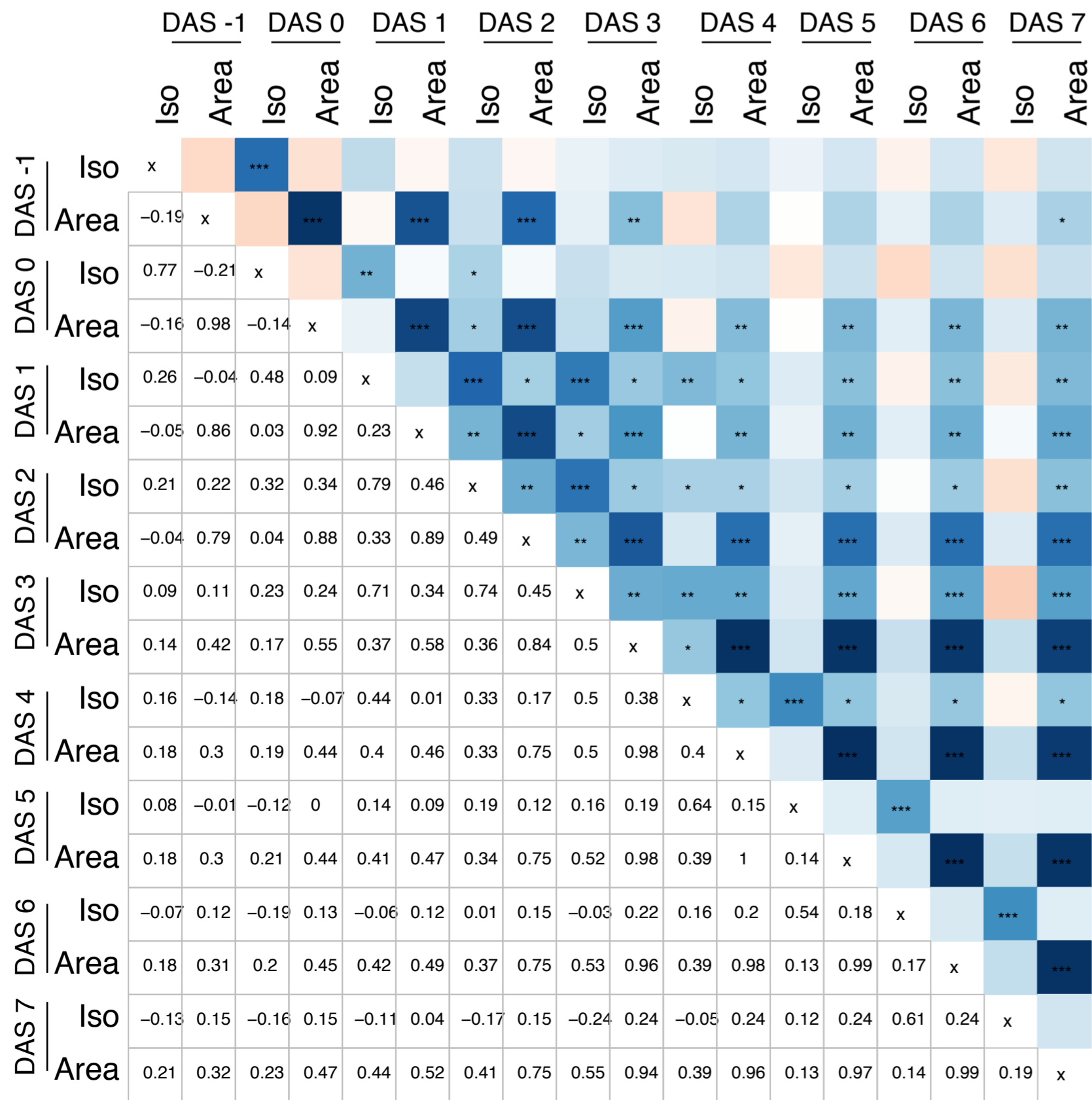

# D9 h heat stress

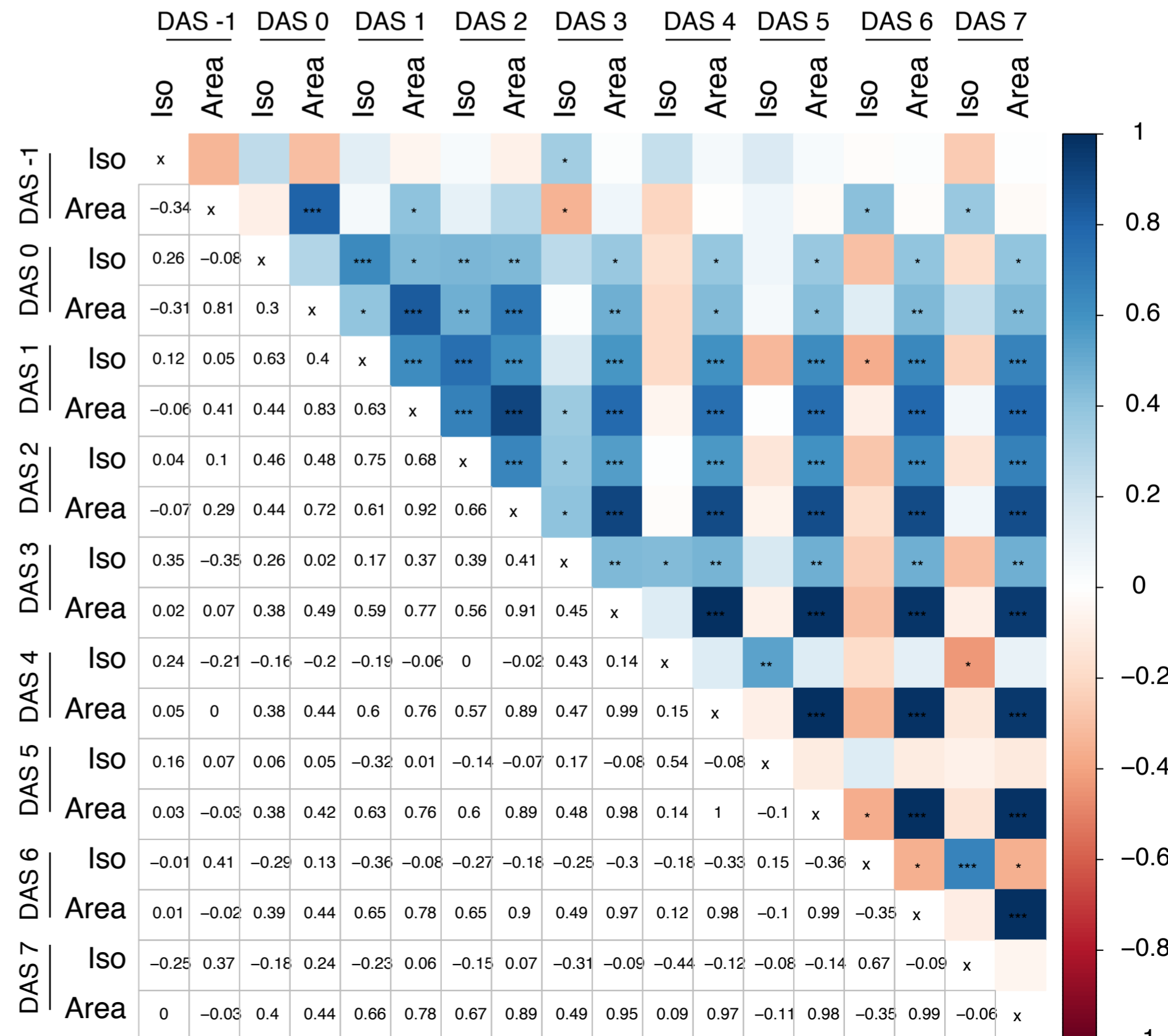

Supplement: Supplementary 8 — Figure S7 Temporal correlation between heat stress-induced changes in rosette isotropy and rosette area. [file 3723916.f8.pdf]

# A0 h heat stress

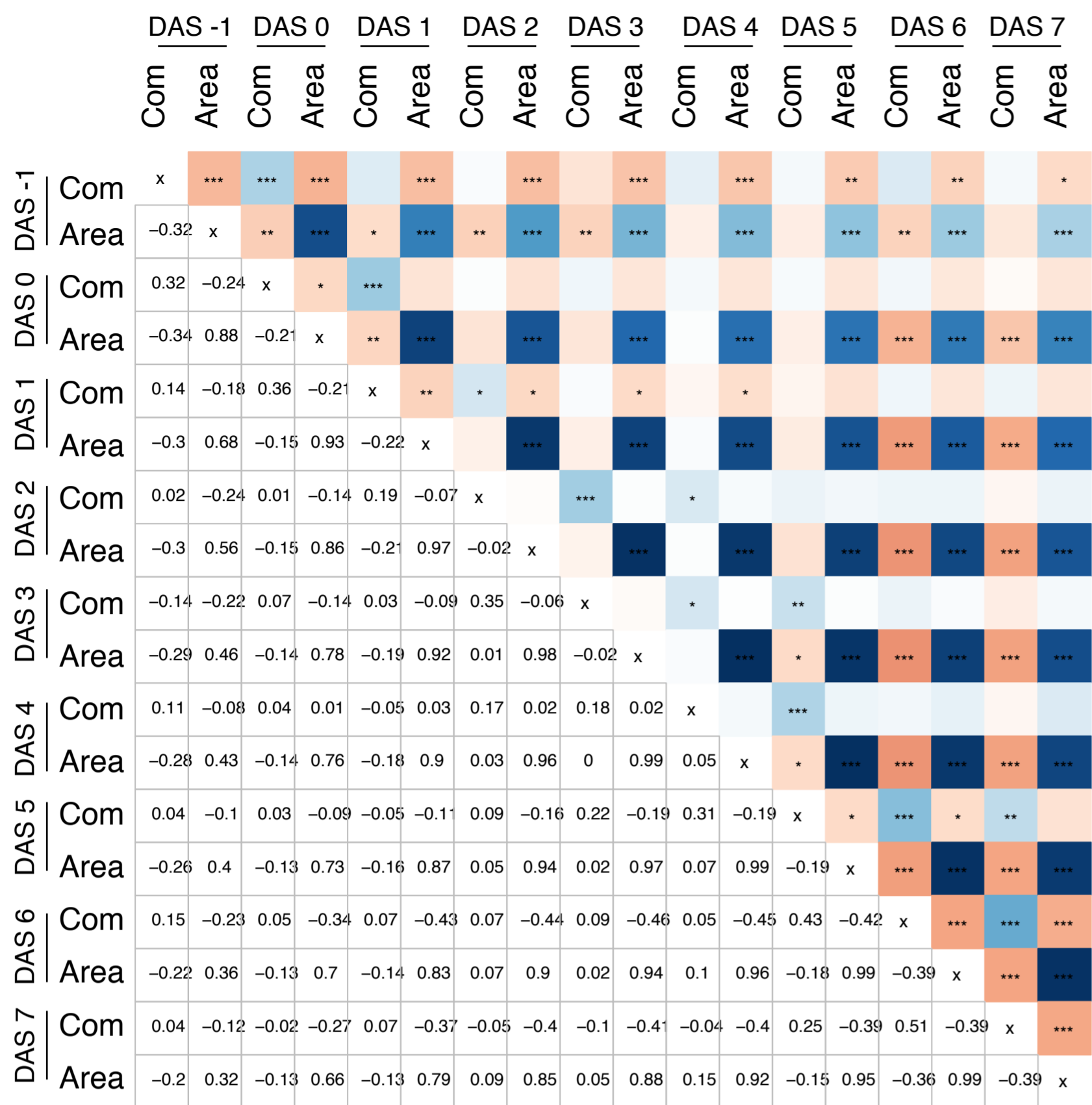

# C6 h heat stress

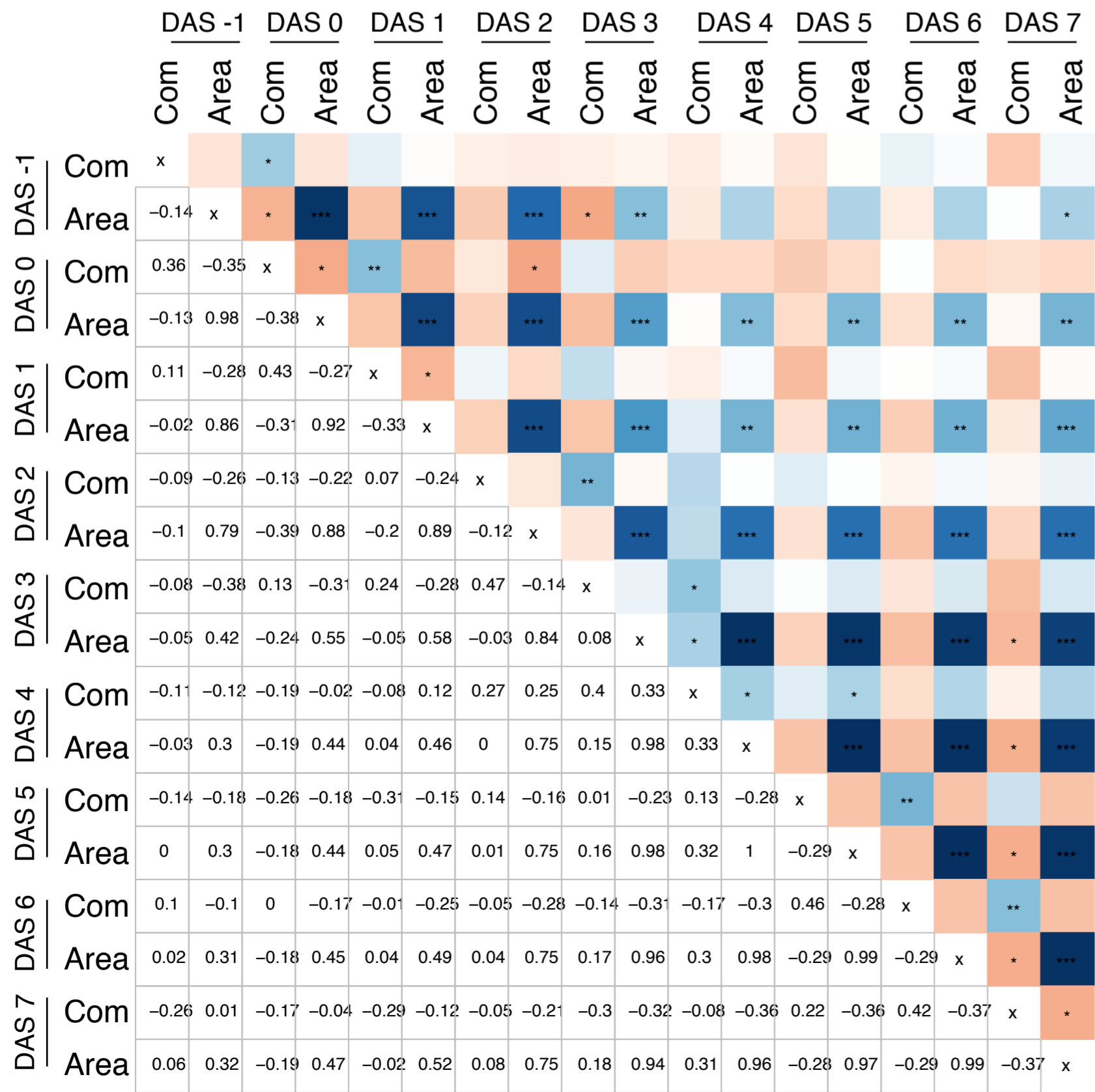

# B3 h heat stress

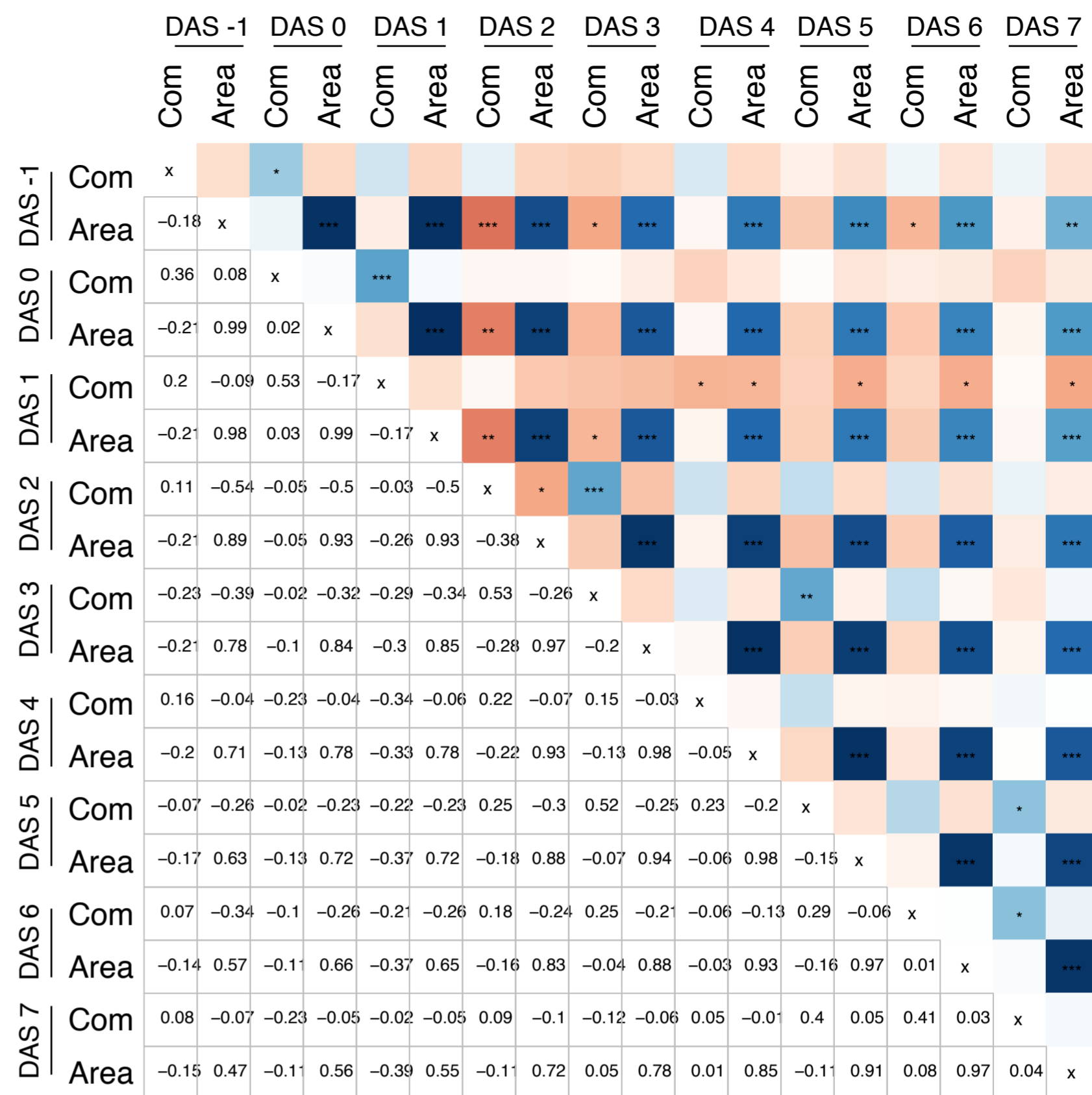

# D9 h heat stress

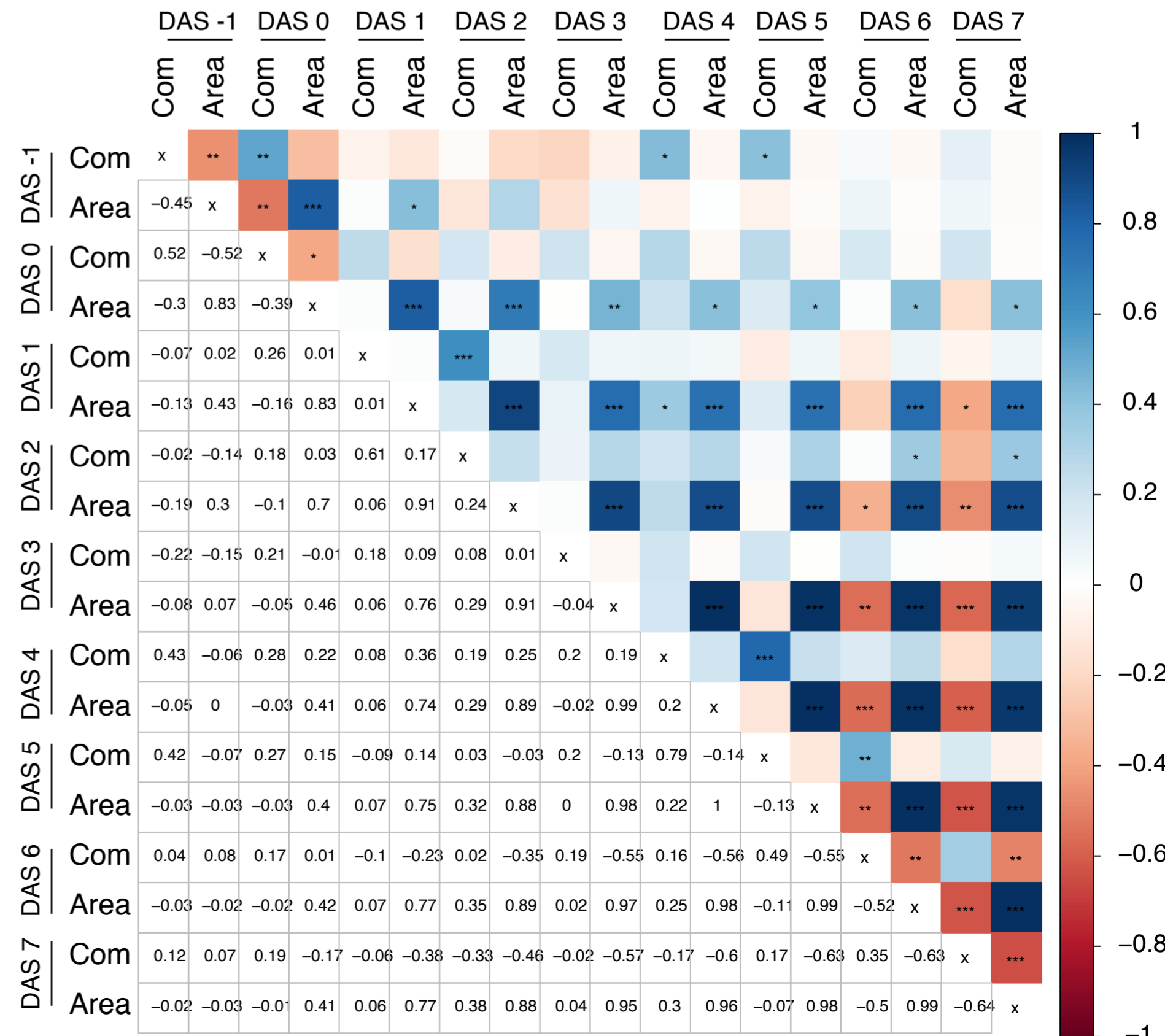

Supplement: Supplementary 9 — Figure S8 Temporal correlation between heat stress-induced changes in rosette compactness and rosette area. [file 3723916.f9.pdf]

# A0 h heat stress

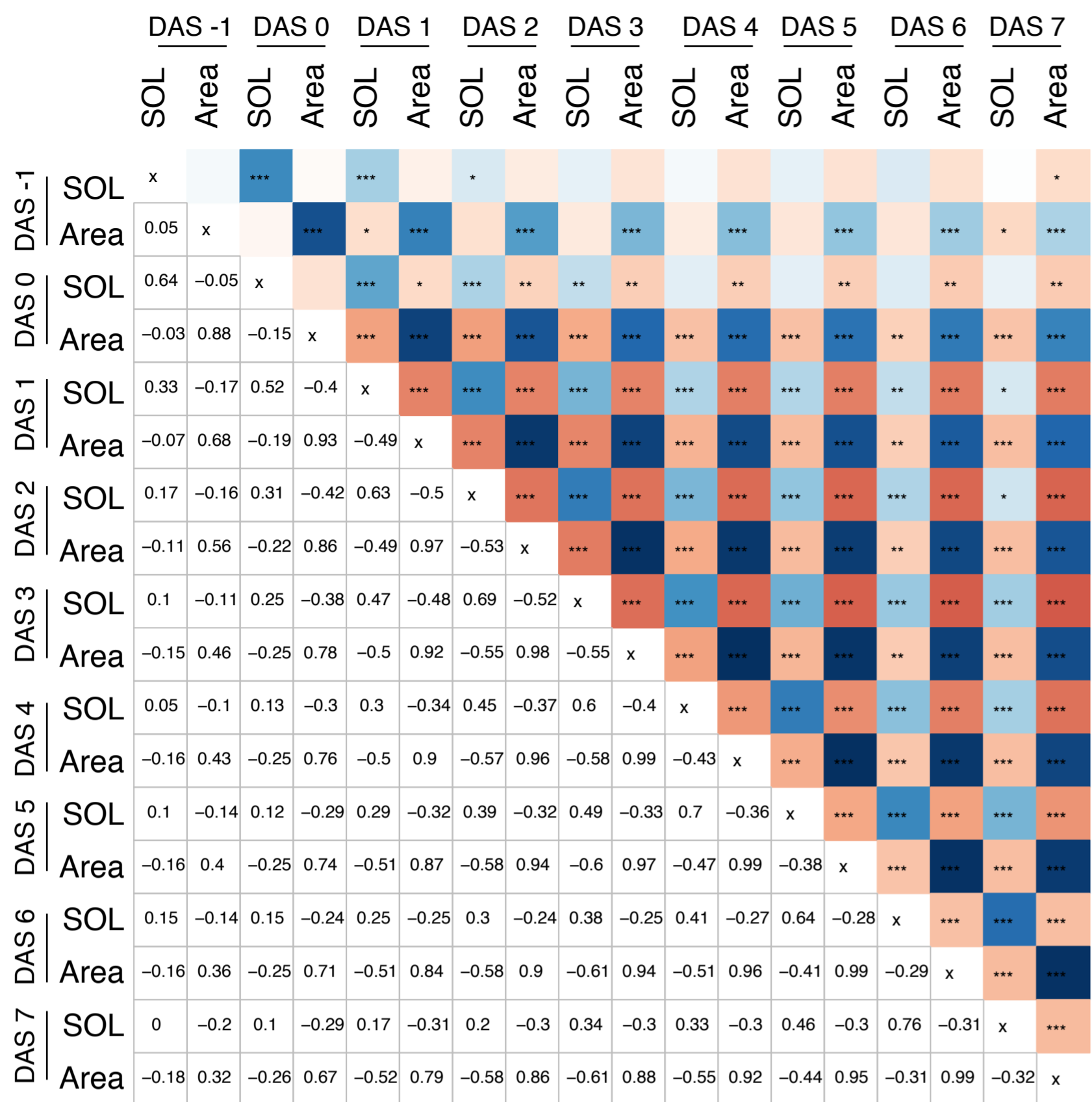

# C6 h heat stress

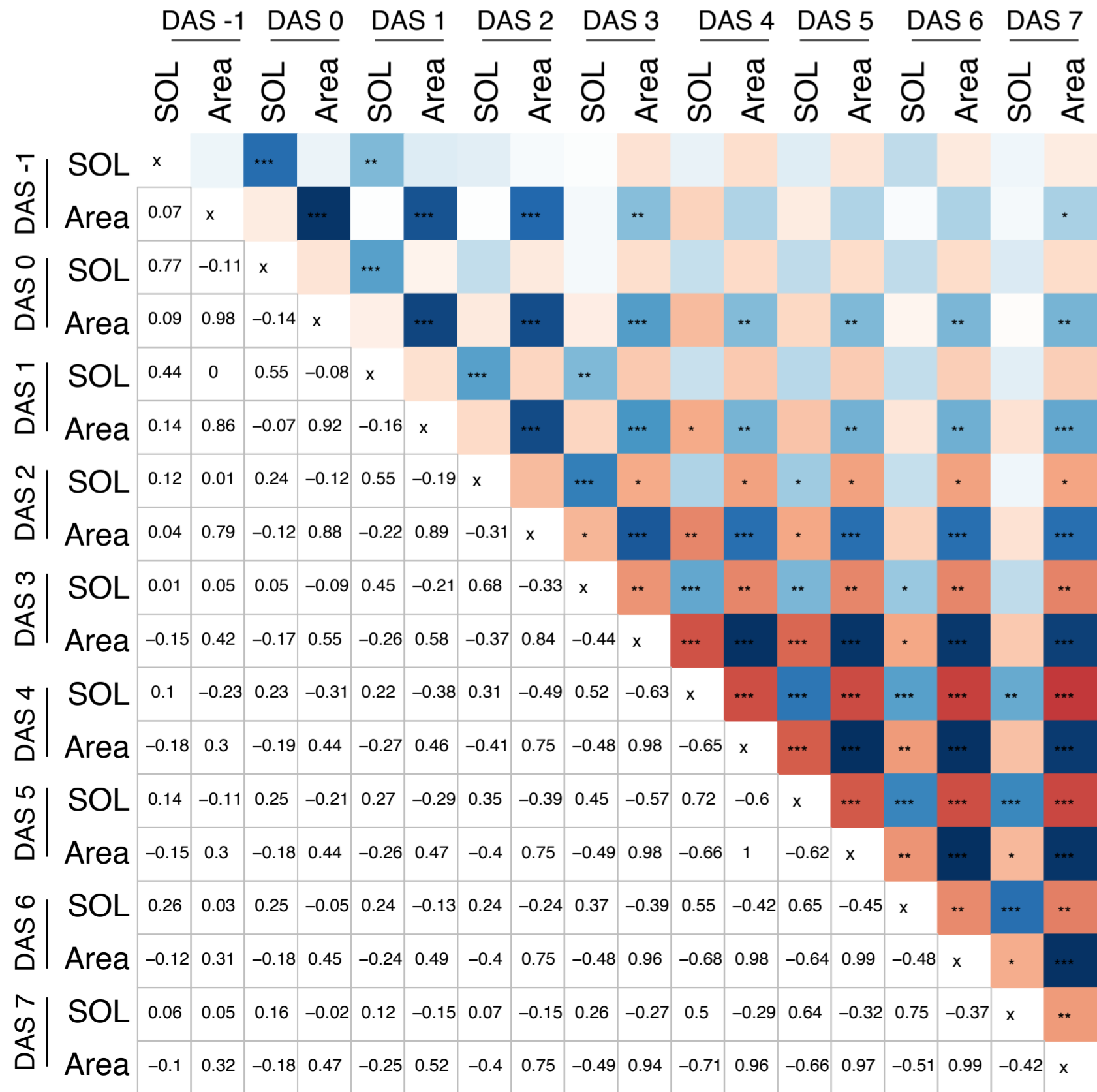

# B3 h heat stress

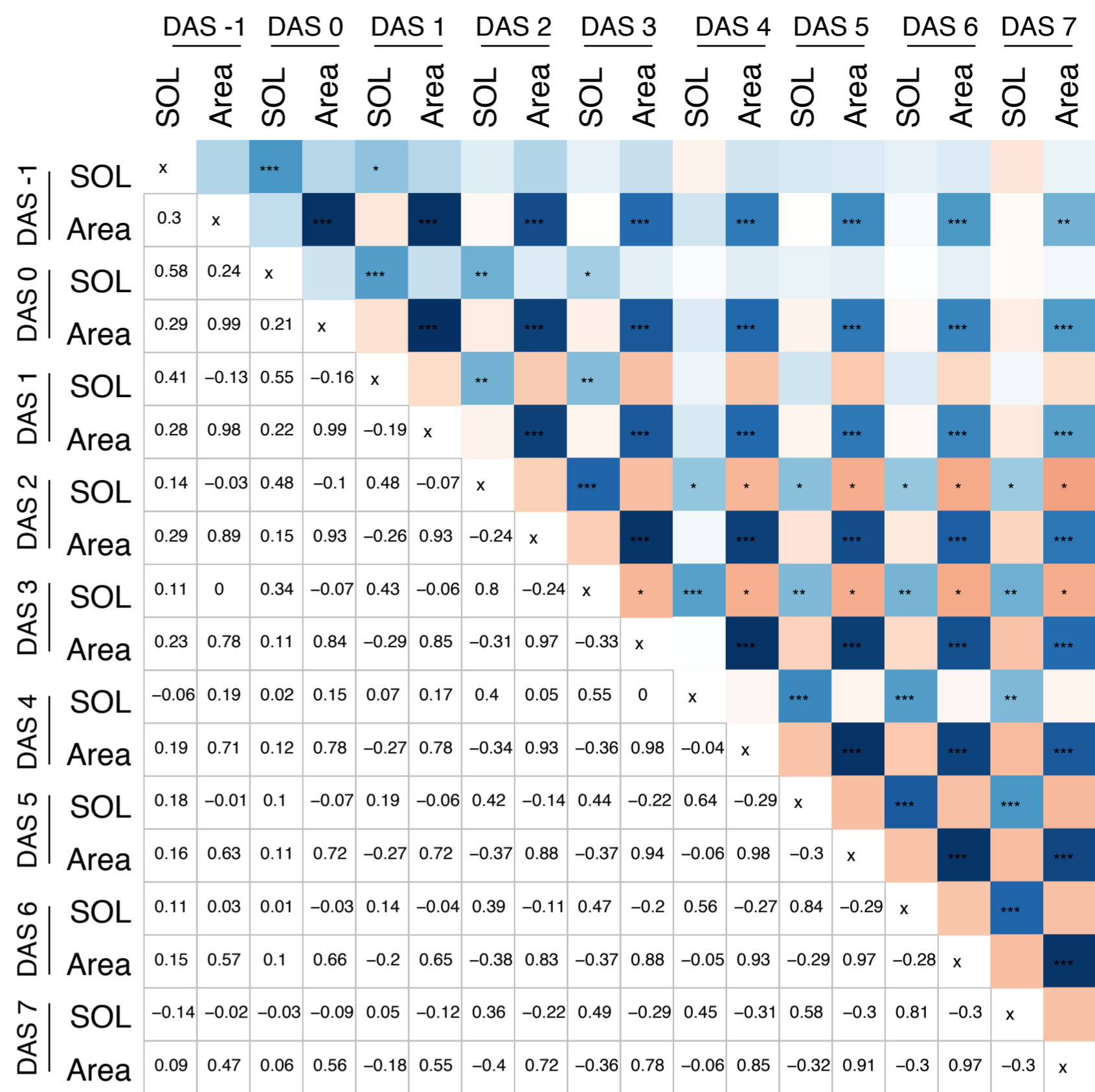

# D9 h heat stress

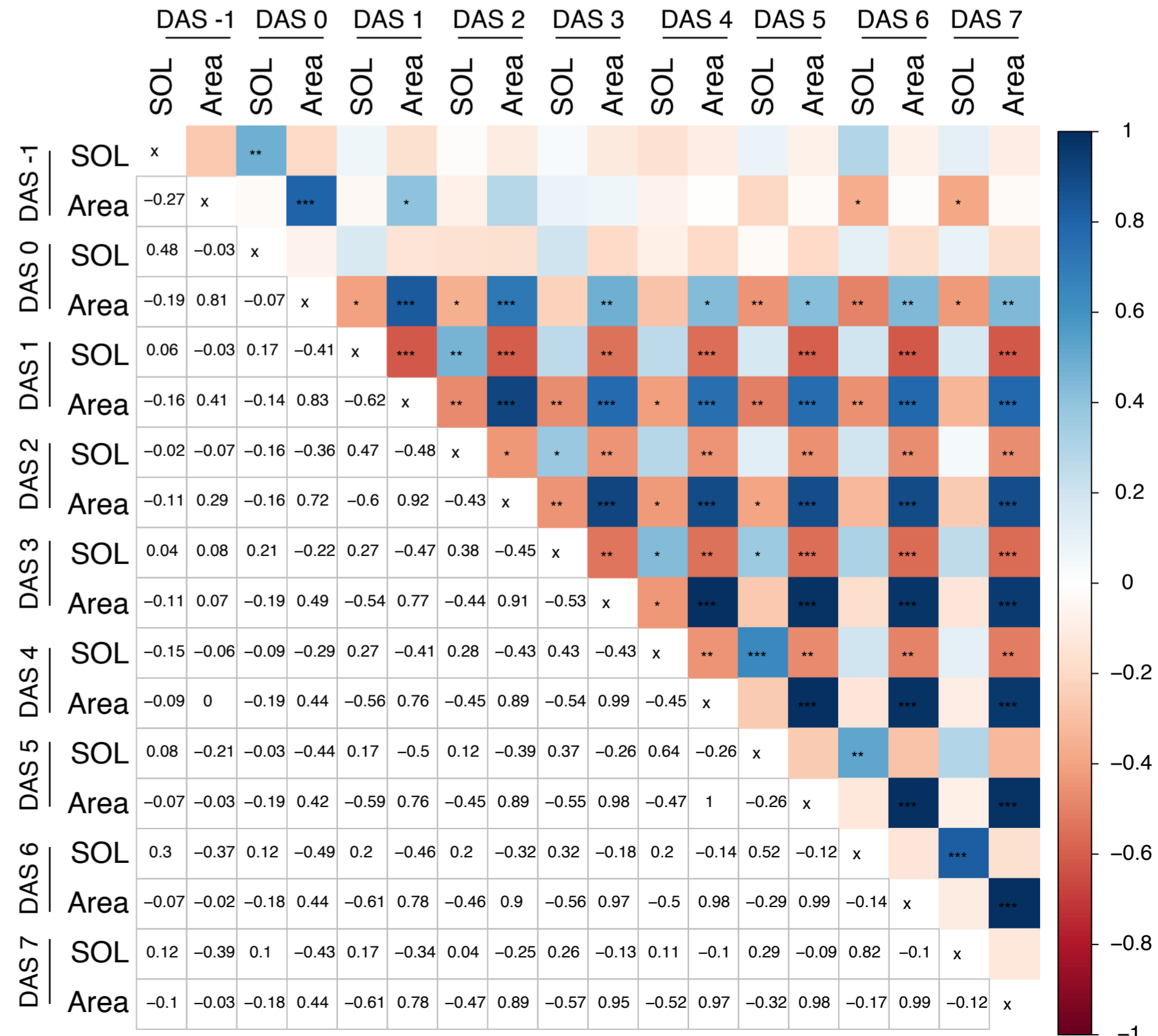

Supplement: Supplementary 10 — Figure S9 Temporal correlation between heat stress-induced changes in slenderness of leaves and rosette area. [file 3723916.f10.pdf]

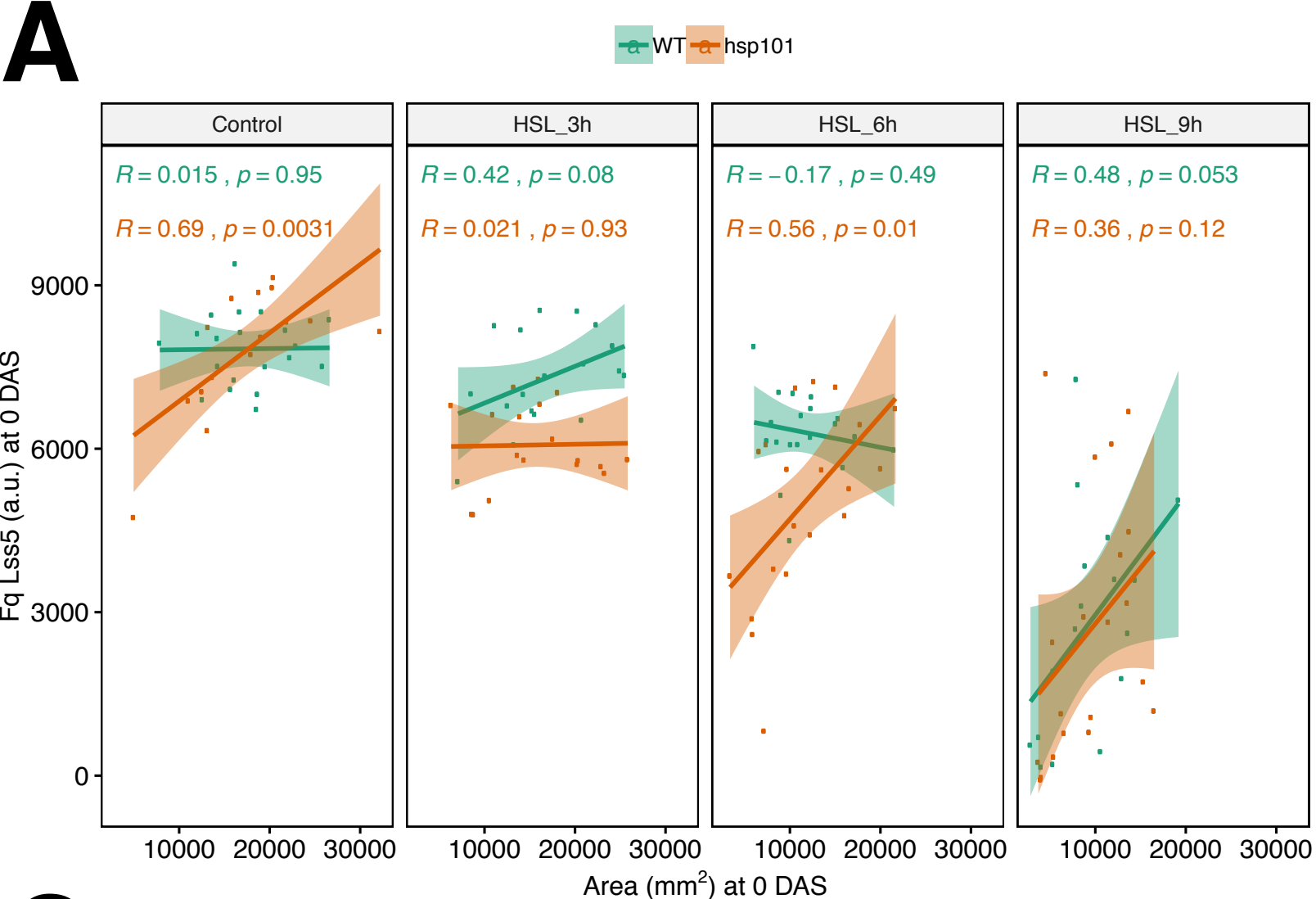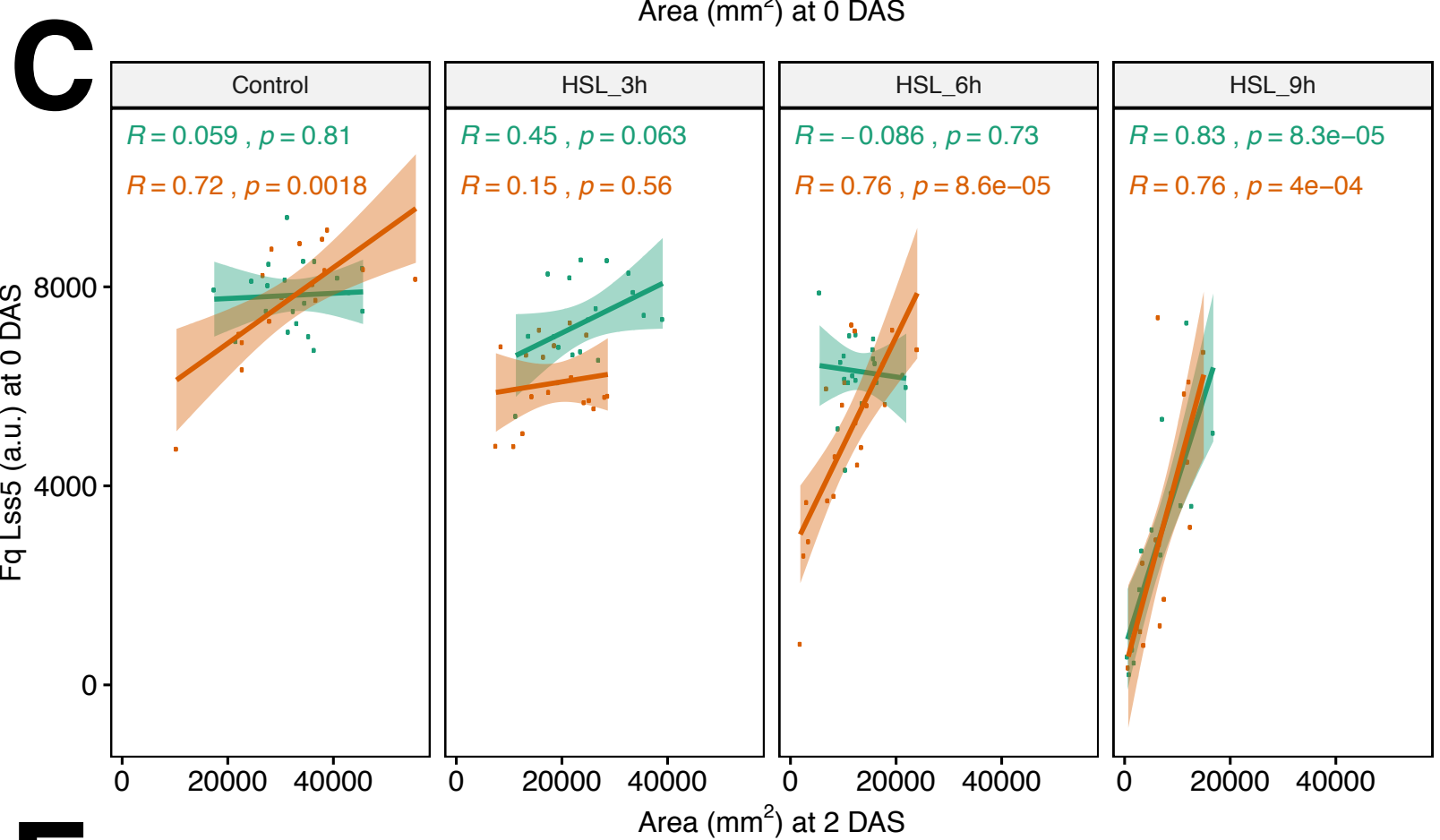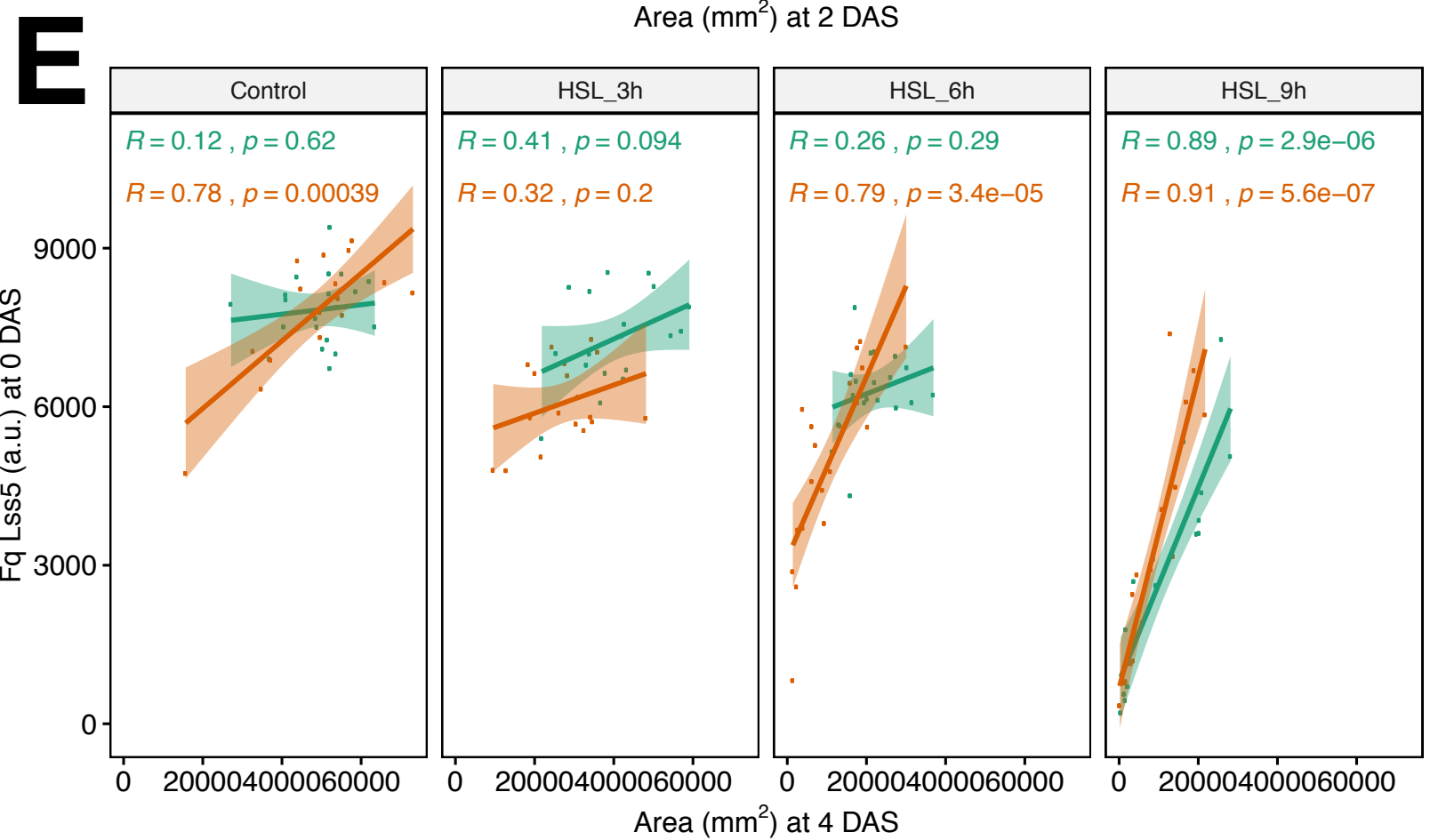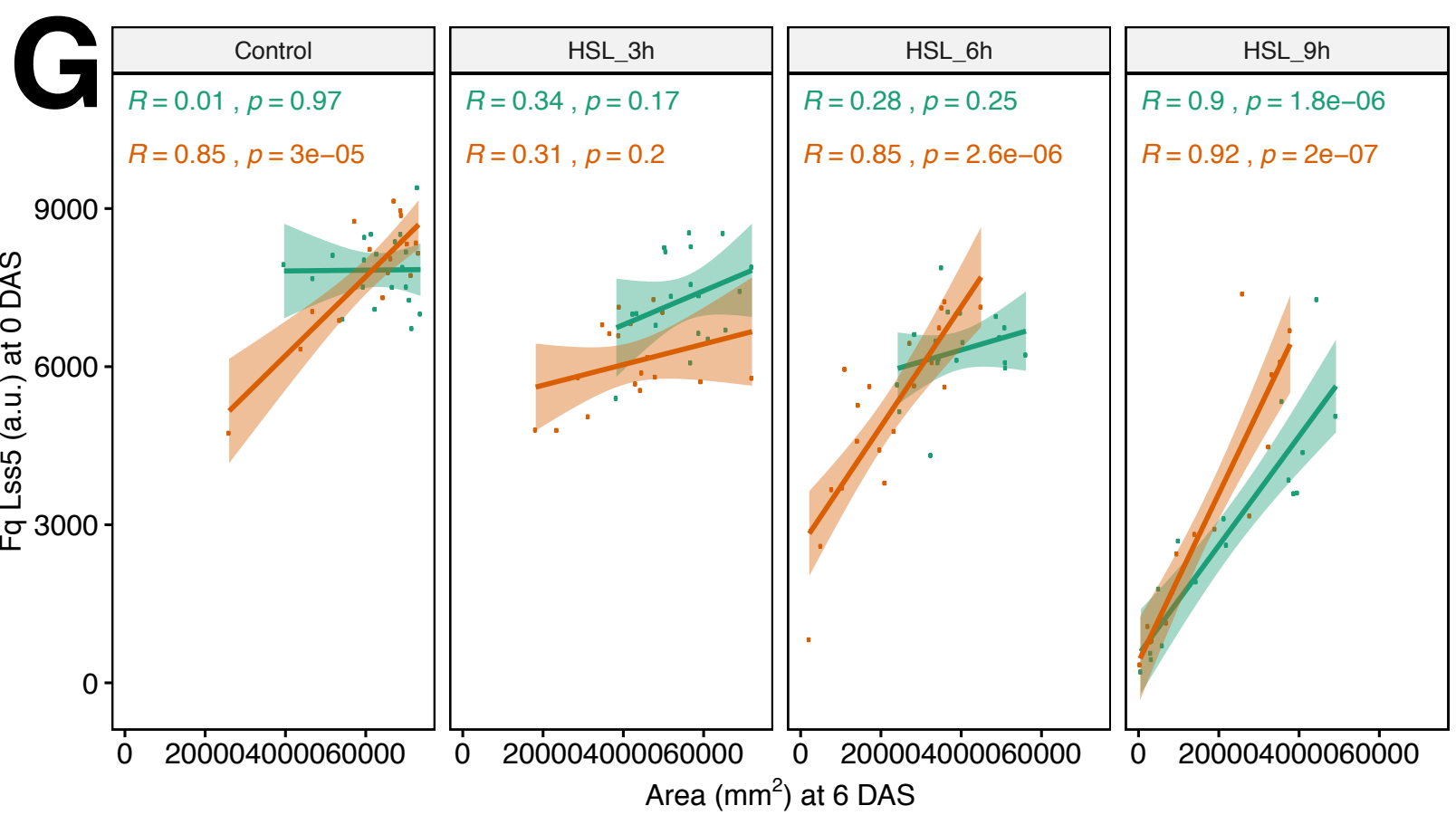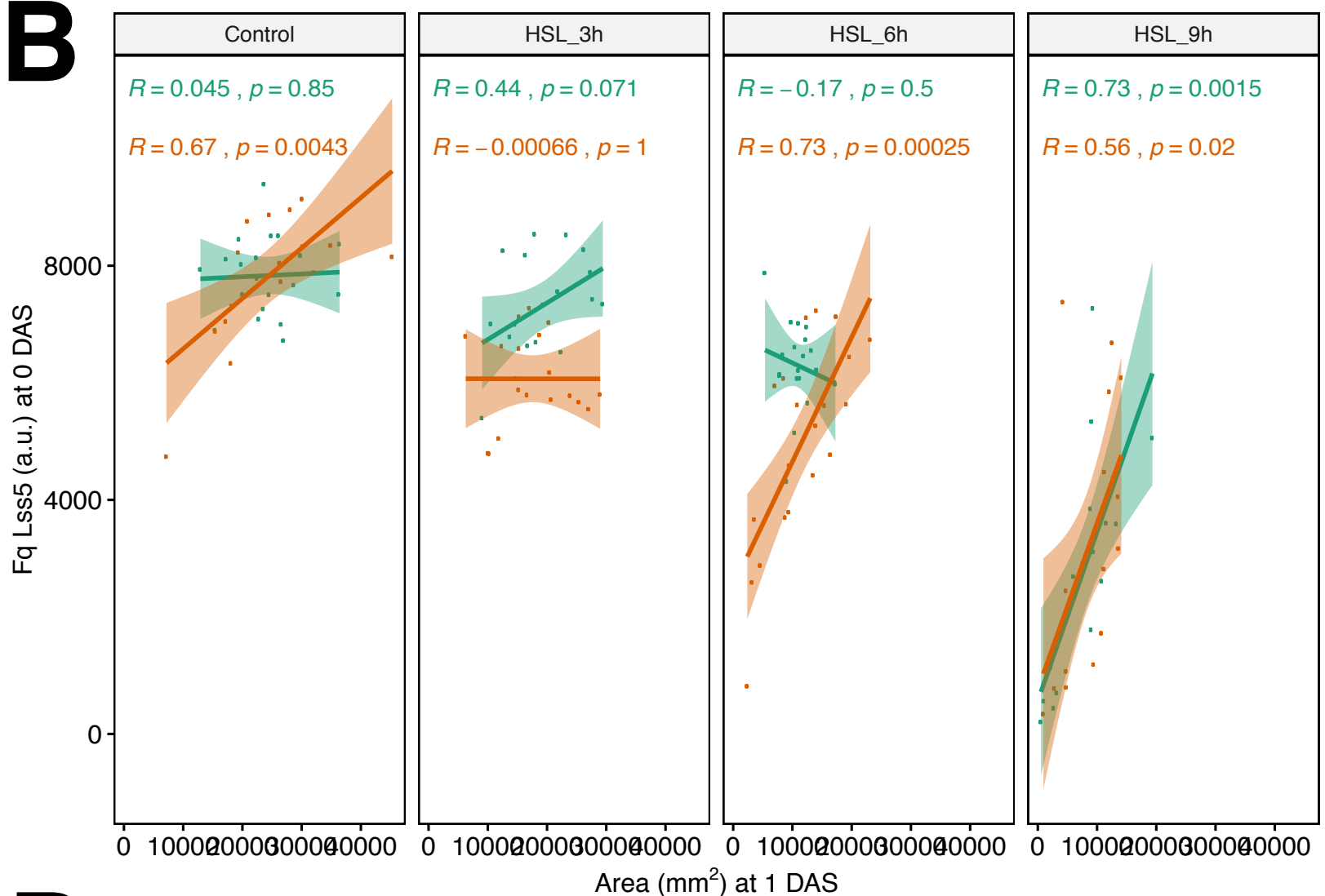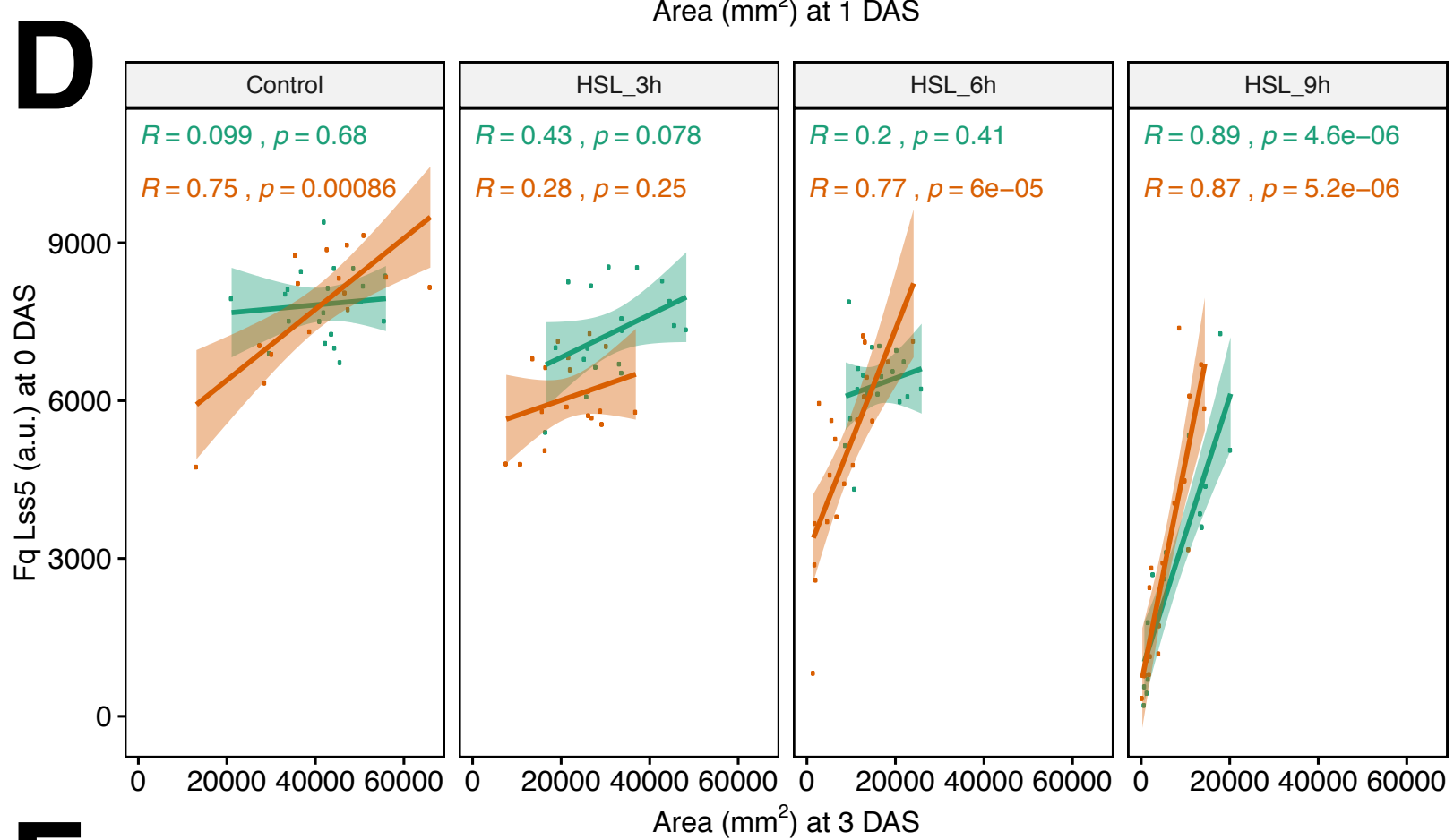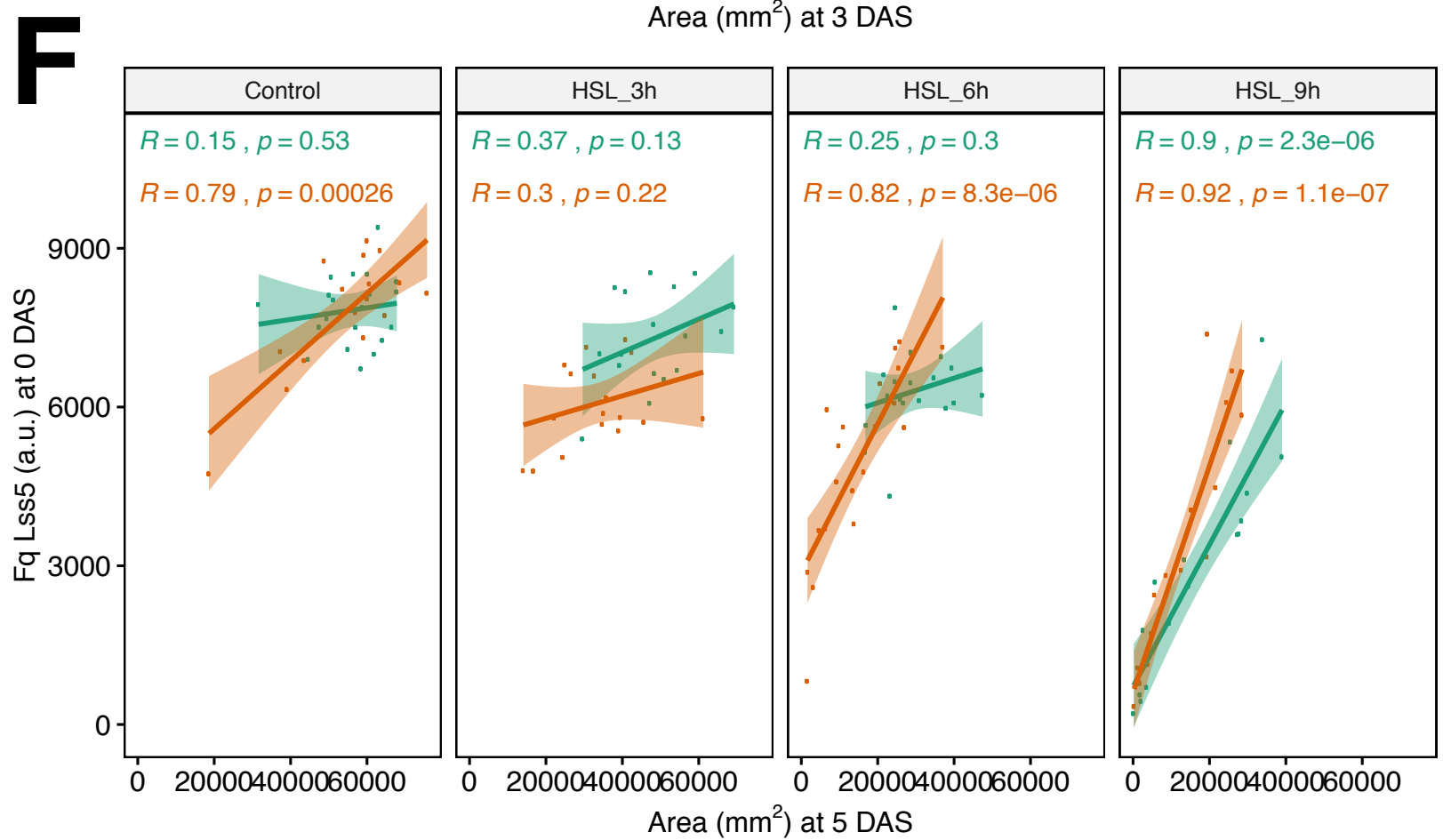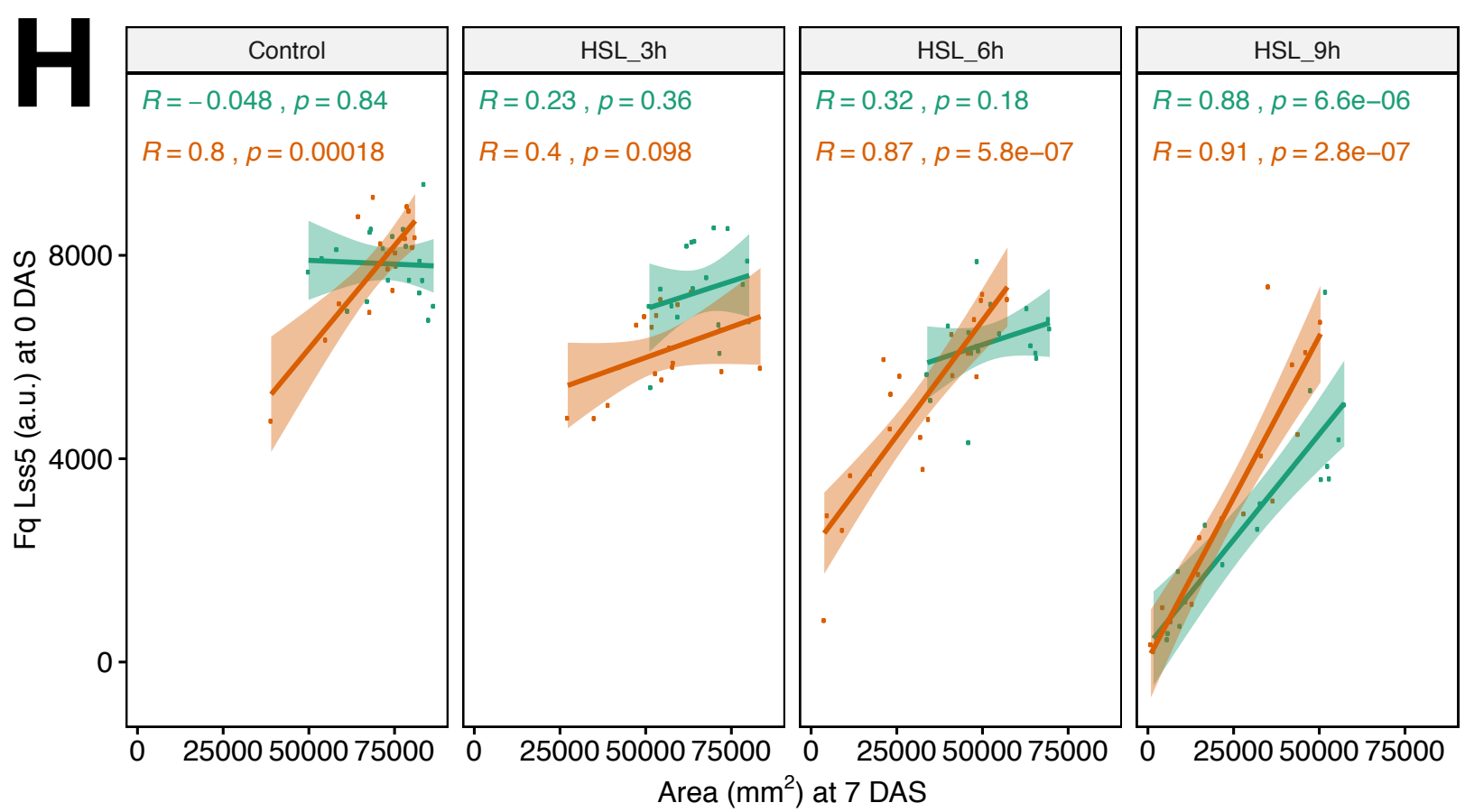

Supplement: Supplementary 11 — Figure S10 Heat stress-induced reduction in Fq at 0 DAS indicates heat susceptibility. [file 3723916.f11.pdf]

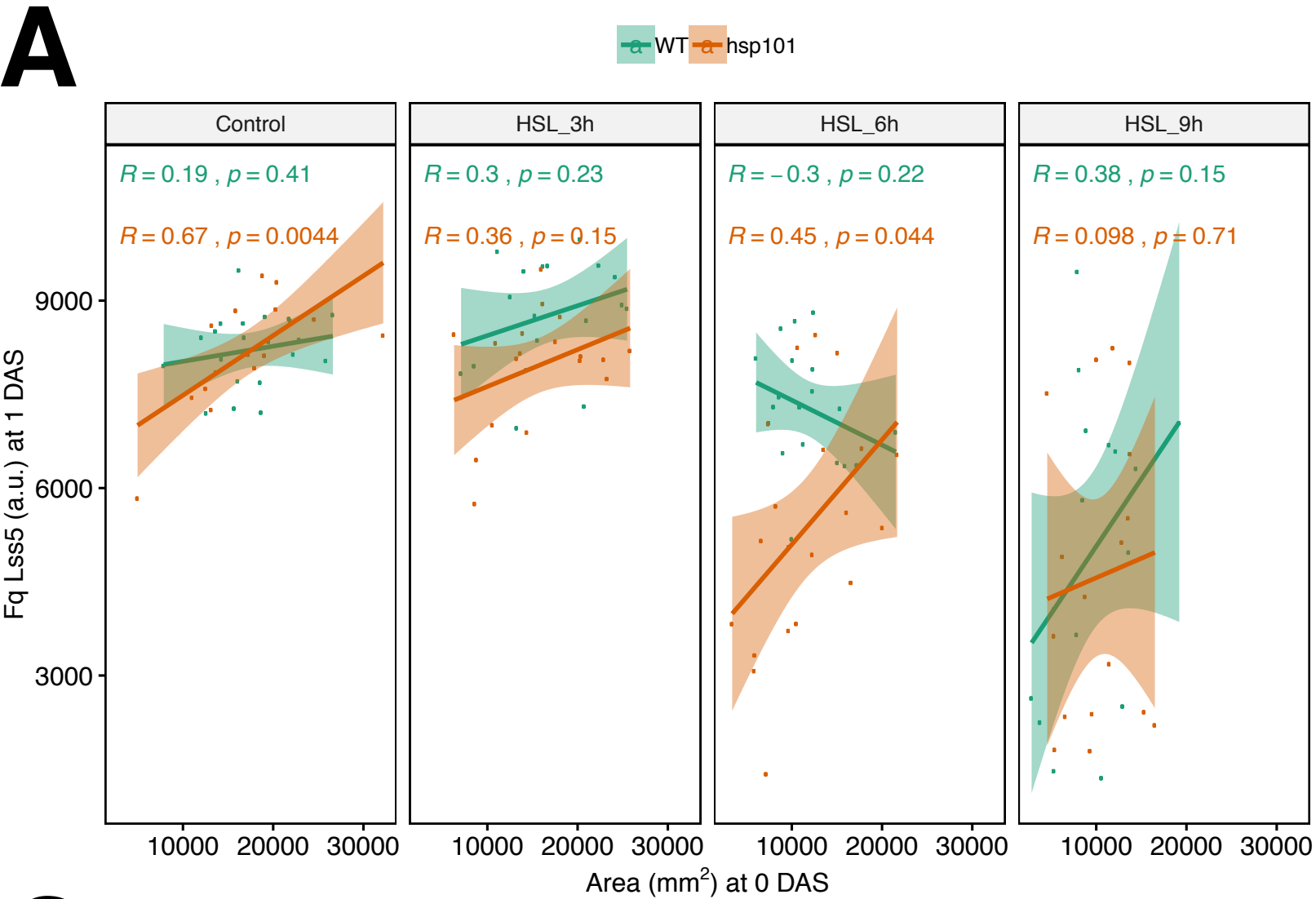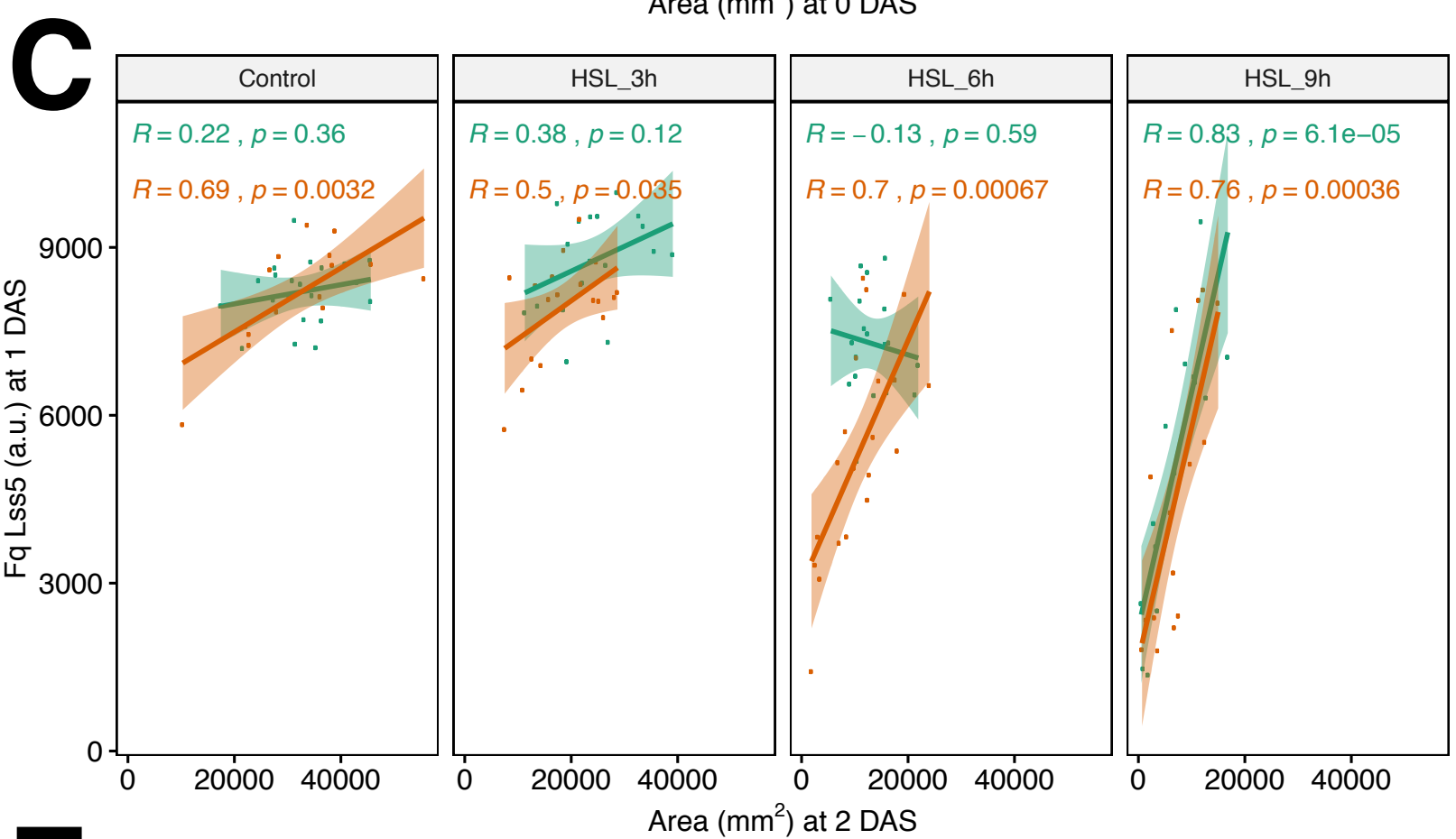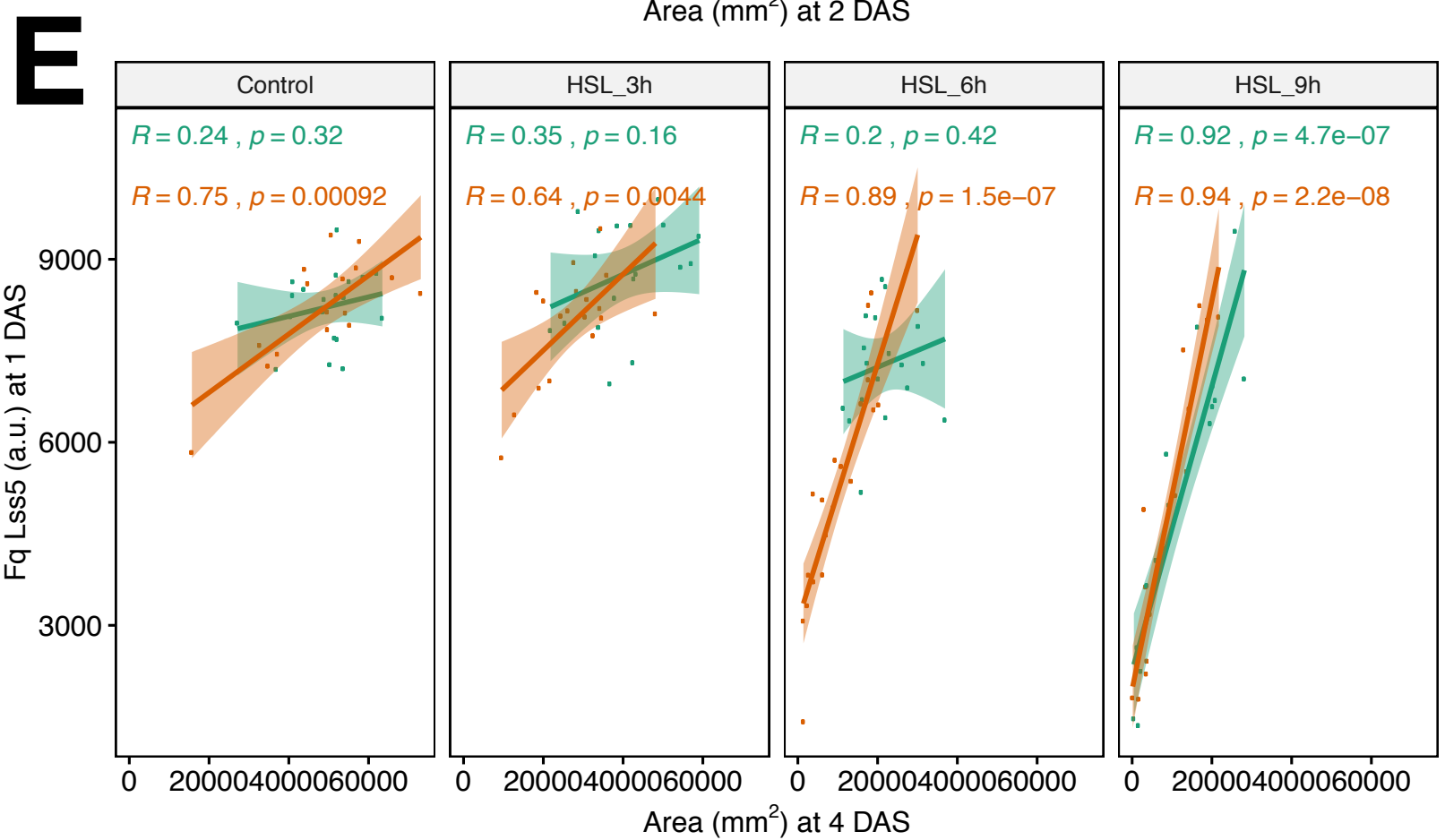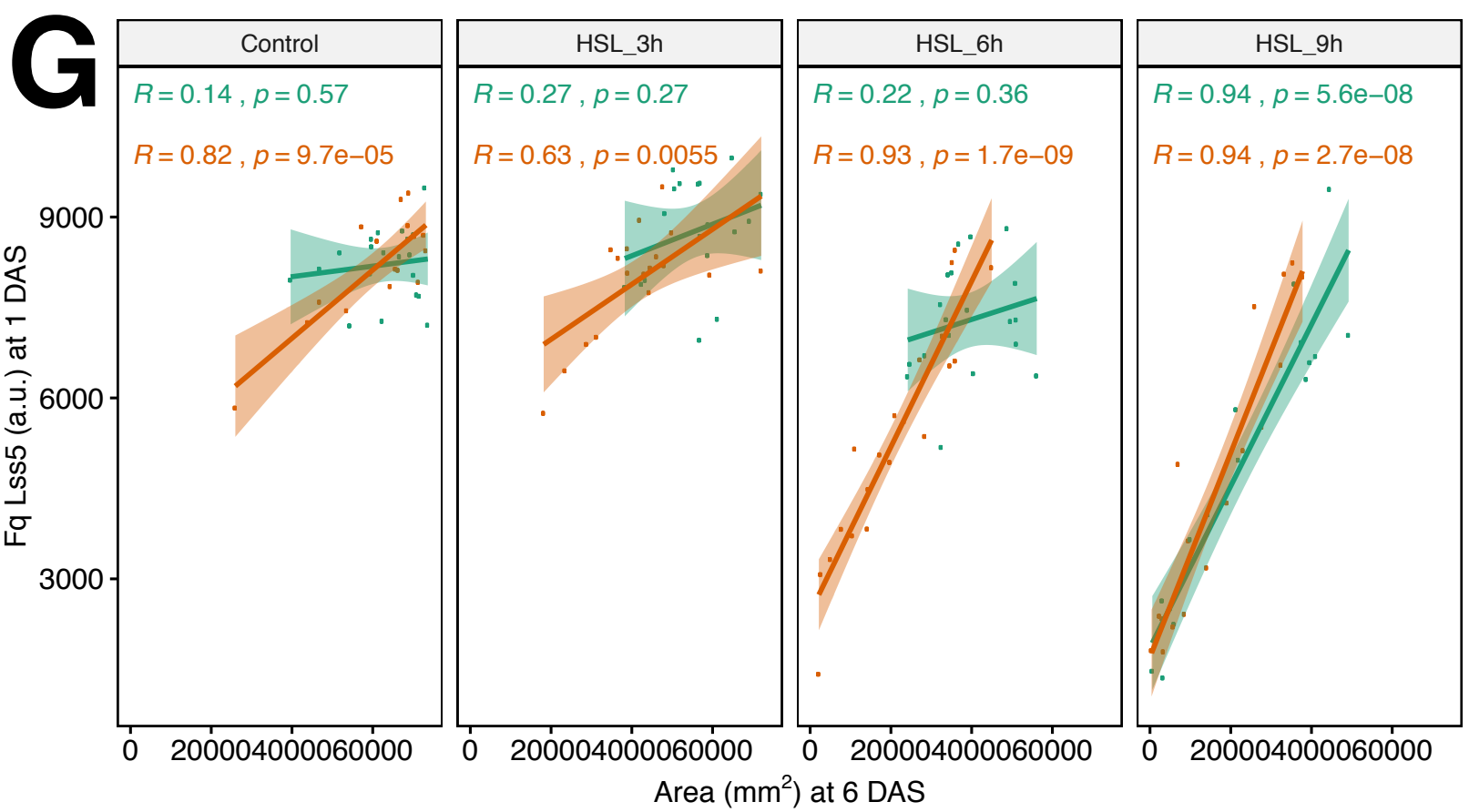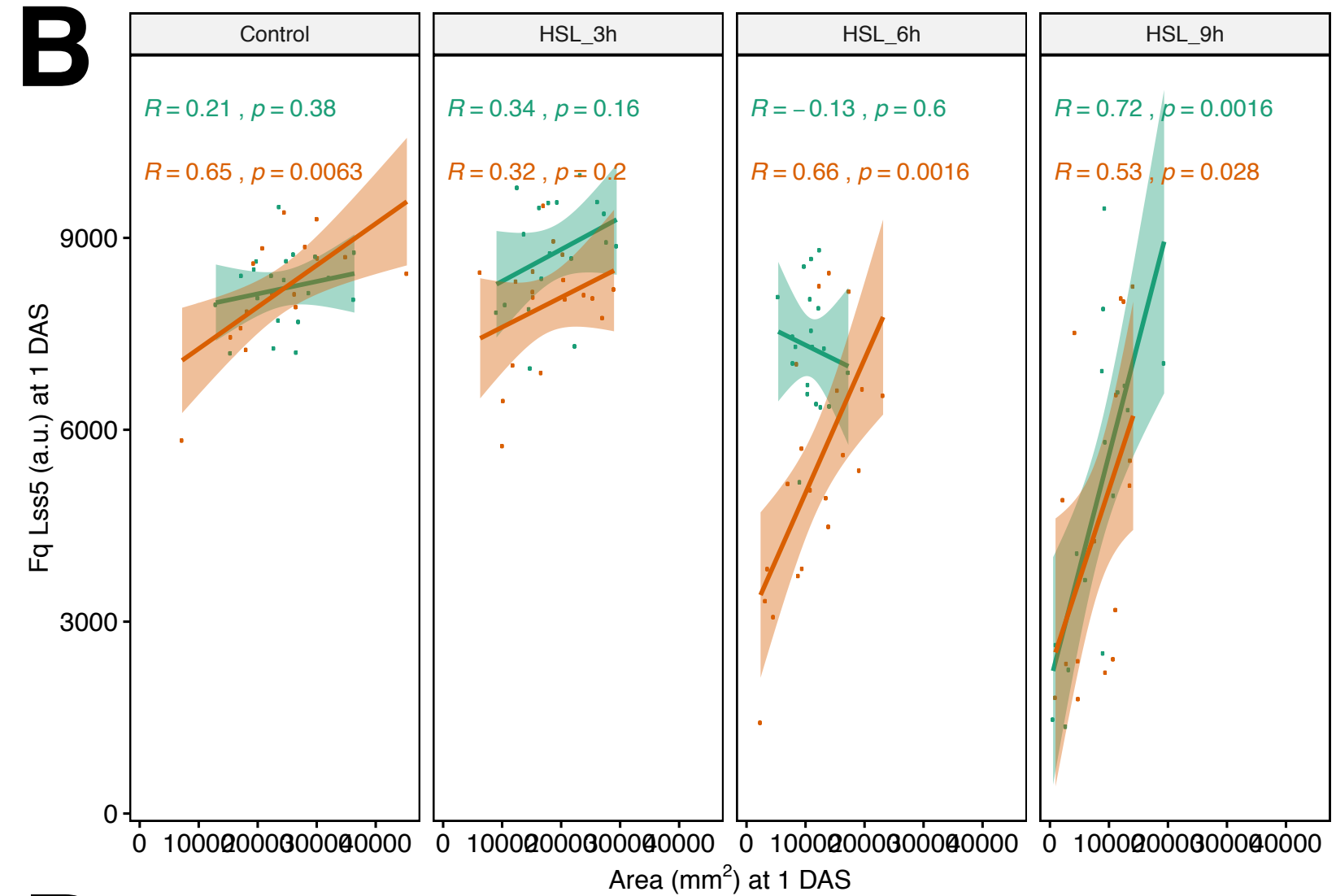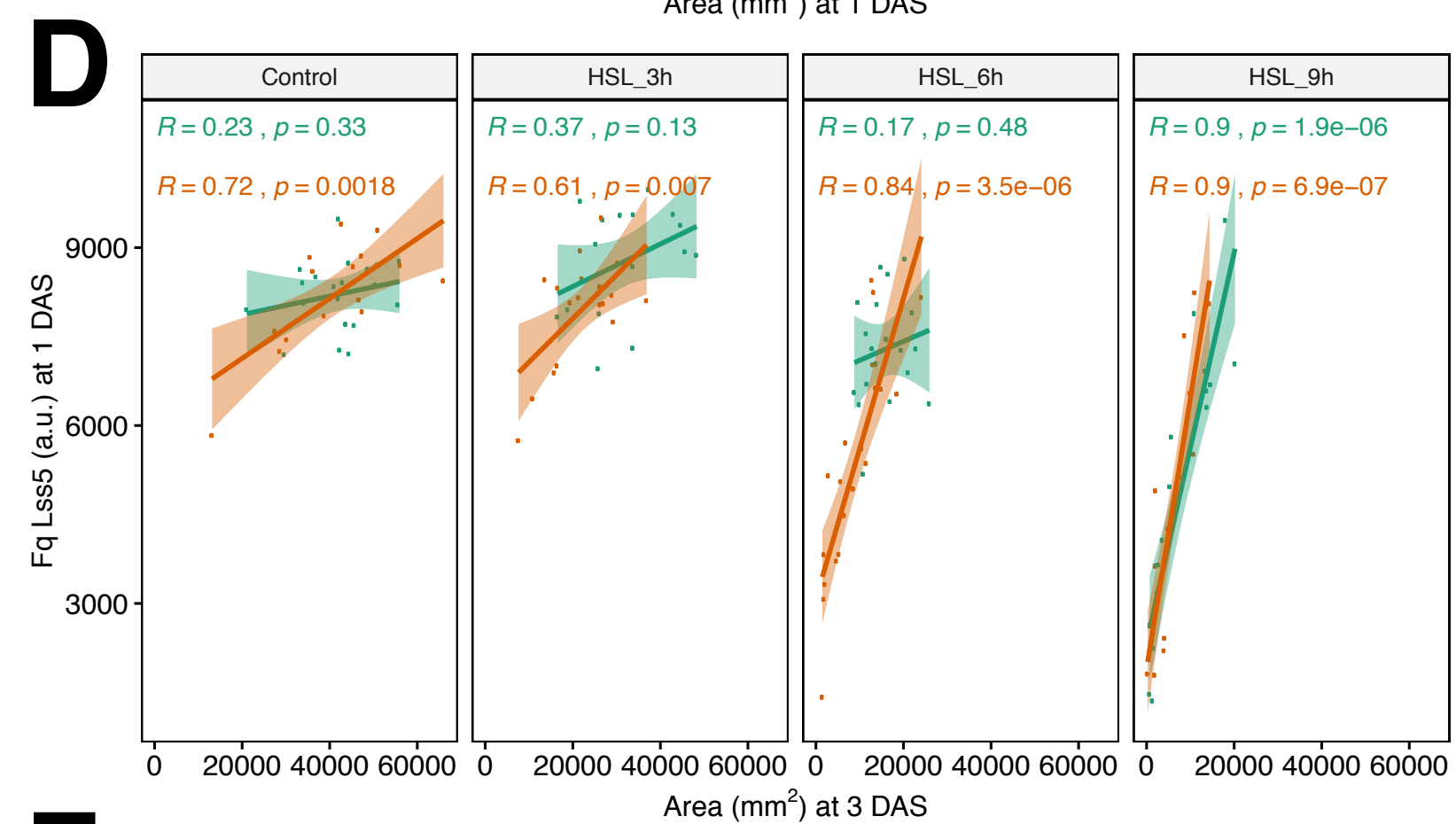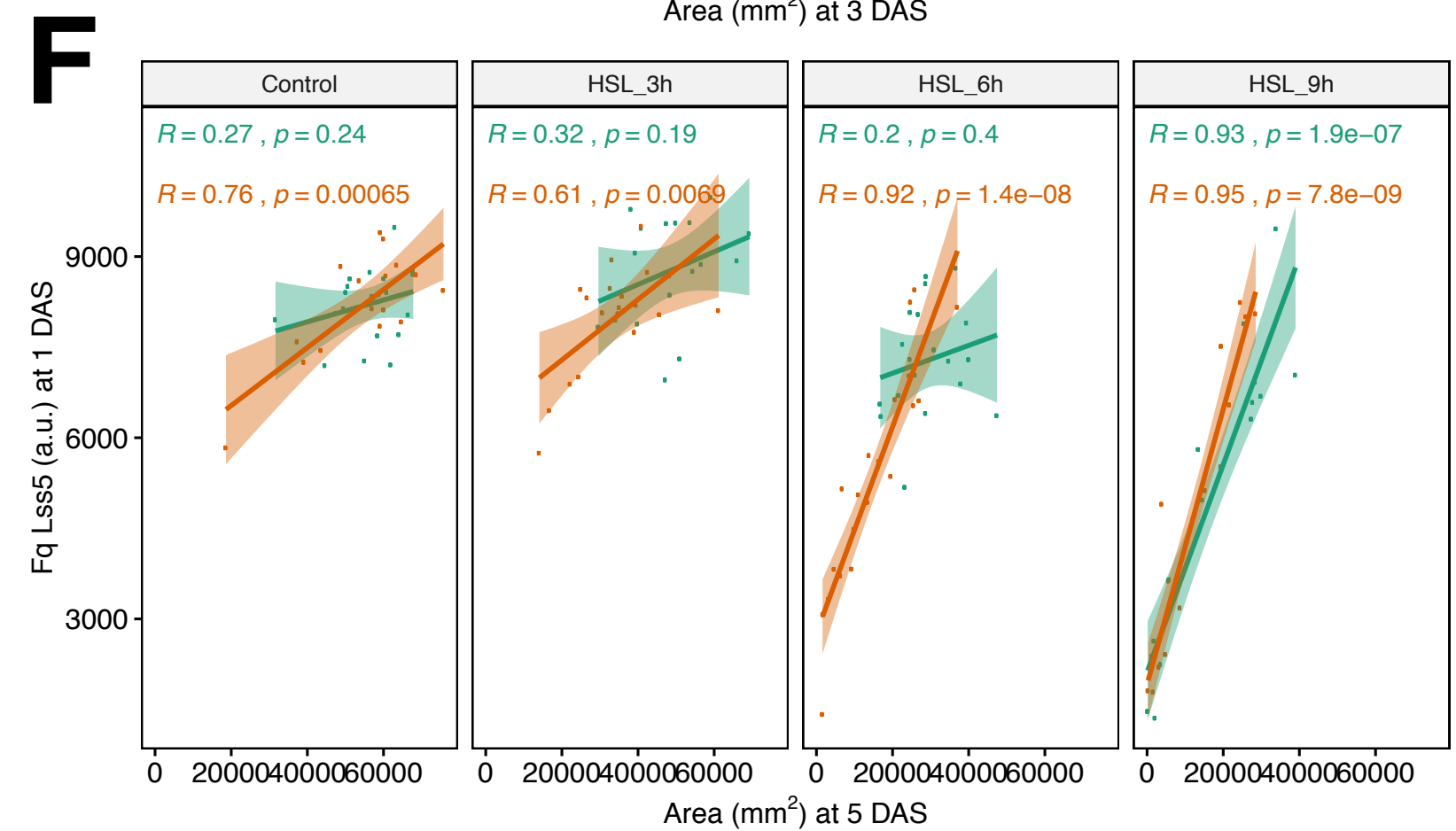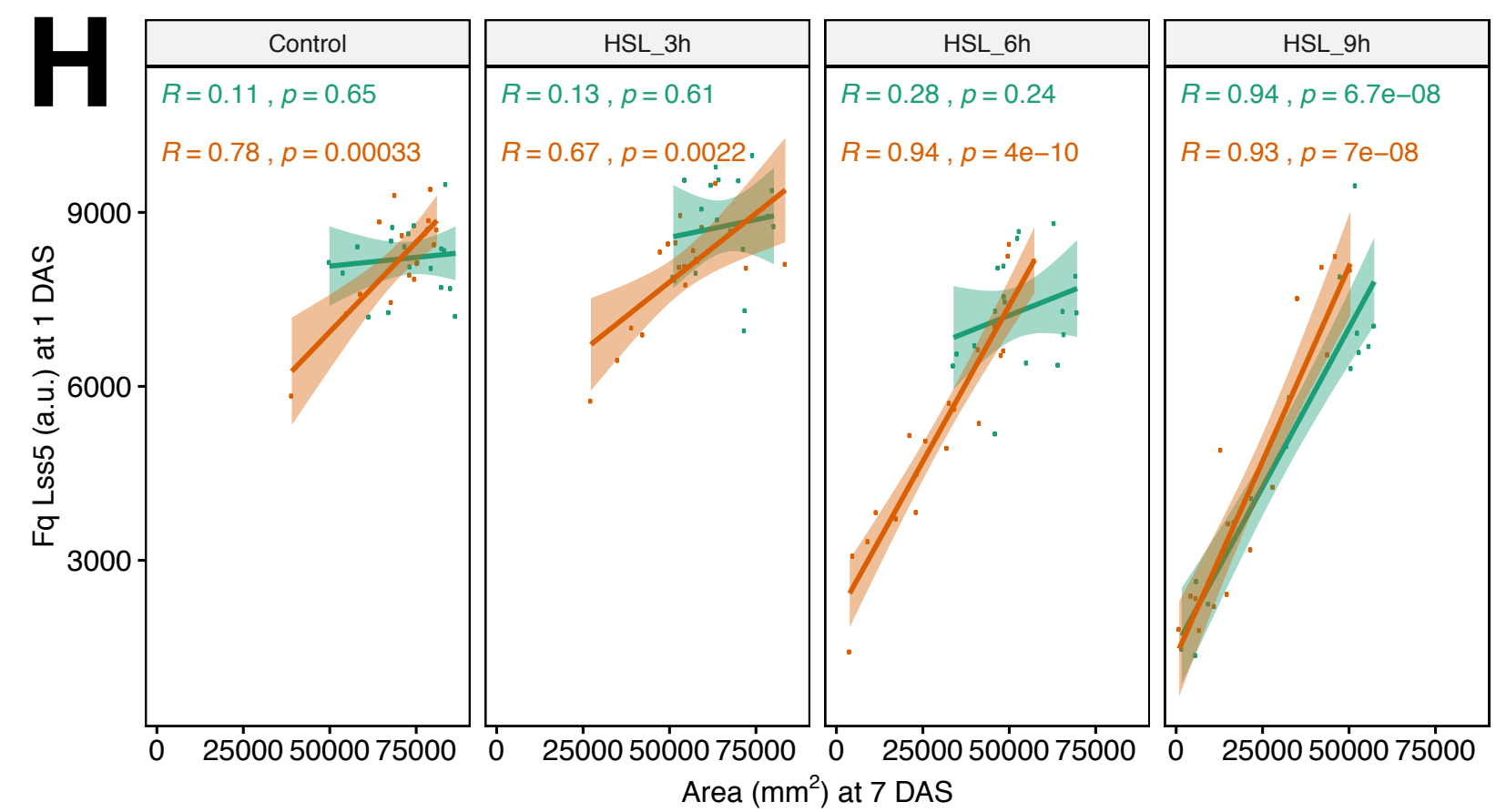

Supplement: Supplementary 12 — Figure S11 Heat-stress induced reduction in Fq at 1 DAS indicates heat susceptibility. [file 3723916.f12.pdf]
